# The *In Vitro* Replication Cycle of Achromobacter xylosoxidans and Identification of Virulence Genes Associated with Cytotoxicity in Macrophages

**DOI:** 10.1128/spectrum.02083-22

**Published:** 2022-07-20

**Authors:** Adam M. Pickrum, Molly O. Riegert, Clive Wells, Kenneth Brockman, Dara W. Frank

**Affiliations:** a Department of Microbiology and Immunology, Medical College of Wisconsin, Milwaukee, Wisconsin, USA; b Department of Cell Biology, Neurobiology and Anatomy, Medical College of Wisconsin, Milwaukee, Wisconsin, USA; Centre National de la Recherche Scientifique, Aix-Marseille Université

**Keywords:** *Achromobacter*, RTX, cytotoxicity, pathogenesis, cystic fibrosis

## Abstract

Achromobacter xylosoxidans is an opportunistic pathogen implicated in a wide variety of human infections including the ability to colonize the lungs of cystic fibrosis (CF) patients. The role of A. xylosoxidans in human pathology remains controversial due to the lack of optimized *in vitro* and *in vivo* model systems to identify and test bacterial gene products that promote a pathological response. We have previously identified macrophages as a target host cell for A. xylosoxidans-induced cytotoxicity. By optimizing our macrophage infection model, we determined that A. xylosoxidans enters macrophages and can reside within a membrane bound vacuole for extended periods of time. Intracellular replication appears limited with cellular lysis preceding an enhanced, mainly extracellular replication cycle. Using our optimized *in vitro* model system along with transposon mutagenesis, we identified 163 genes that contribute to macrophage cytotoxicity. From this list, we characterized a giant RTX adhesin encoded downstream of a type one secretion system (T1SS) that mediates bacterial binding and entry into host macrophages, an important first step toward cellular toxicity and inflammation. The RTX adhesin is encoded by other human isolates and is recognized by antibodies present in serum isolated from CF patients colonized by A. xylosoxidans, indicating this virulence factor is produced and deployed *in vivo*. This study represents the first characterization of A. xylosoxidans replication during infection and identifies a variety of genes that may be linked to virulence and human pathology.

**IMPORTANCE** Patients affected by CF develop chronic bacterial infections characterized by inflammatory exacerbations and tissue damage. Advancements in sequencing technologies have broadened the list of opportunistic pathogens colonizing the CF lung. A. xylosoxidans is increasingly recognized as an opportunistic pathogen in CF, yet our understanding of the bacterium as a contributor to human disease is limited. Genomic studies have identified potential virulence determinants in A. xylosoxidans isolates, but few have been mechanistically studied. Using our optimized *in vitro* cell model, we identified and characterized a bacterial adhesin that mediates binding and uptake by host macrophages leading to cytotoxicity. A subset of serum samples from CF patients contains antibodies that recognize the RTX adhesion, suggesting, for the first time, that this virulence determinant is produced *in vivo*. This work furthers our understanding of A. xylosoxidans virulence factors at a mechanistic level.

## INTRODUCTION

Members of the *Achromobacter* genus are Gram-negative bacilli (1) that can be found widely distributed in nature and are recognized as emerging opportunistic pathogens (2). Although potentially causing a variety of infections, *Achromobacter* spp. are routinely isolated from patients affected by cystic fibrosis (CF) (3). CF leads to the development of chronic opportunistic bacterial infections that result in a deleterious inflammatory response, further damaging fragile airways. Studies suggest that A. xylosoxidans (*Ax*) is the most common *Achromobacter* species recovered from the lungs of CF patients, followed by *A. ruhlandii* (4, 5).

While considered opportunists, *Achromobacter* spp. possess properties consistent with pathogens including the ability to colonize human lungs (6–9) to be transmitted from environmental reservoirs (10) and from patient to patient (11, 12). *Ax* colonization is associated with older CF patients and declining lung function (6, 13–16), and those who undergo lung transplantation immediately develop reinfection (17). A contributing factor that may enhance the retention of *Achromobacter* spp. in the CF lung is the almost universal resistance to a variety of antibiotics including aminoglycosides and cephalosporins (18). A loss of bacterial diversity in CF sputum samples dominated with *Ax* has been reported (19) and may be explained by the ability of *Ax* to outcompete other bacteria in the CF lung environment (20). Finally, the ability to form protective biofilms (21–23) may act in concert with the competitiveness of *Ax* and its intrinsic antimicrobial resistance.

Despite clinical reports of *Achromobacter* spp.*-*mediated infections, a major knowledge gap exists concerning the molecular mechanisms underlying pathogenesis. Sequenced genomes of isolates offer a predictive but untested understanding of potential virulence-related pathways thought to function in toxin secretion, antibiotic resistance, extracellular polysaccharide synthesis, adhesion, and horizontal gene transfer (24–27). Comparative genomic analysis of clonal lineages of *Achromobacter* spp. isolated from five patients with CF demonstrated a cumulative pattern of mutations in genes related to antibiotic resistance efflux pumps, cell wall and capsule biosynthesis and iron acquisition (28). These studies suggest that *Ax* establishes and maintains infections in humans but due to a lack of functional studies, the linkage between colonization and pathology is unclear.

A growing number of studies aim to define pathogenic mechanisms of *Ax*. Data suggest that *Ax* secretes toxins that have protease activity (29) and induce cellular morphological changes along with inflammatory cytokine expression in carcinoma cells (30). *Achromobacter* spp. can prevent adhesion, biofilm formation (31), and inactivate quorum sensing molecules (32) of Pseudomonas aeruginosa, a dominant CF pathogen. Gene deletions in *Ax* strains demonstrated the contribution of two RND-type multidrug efflux systems to antibiotic resistance (33–35). Transposon mutagenesis of *Ax* identified genes involved in biofilm formation (23), but genetic manipulation of *Ax* has been inconsistent due to lack of standardized strains. Our group identified macrophages as a cell type that is susceptible to infection with a subset of *Ax* clinical isolates, including strain GN050 (36). Bacterial uptake induces host cytotoxicity and transcriptional activation of the type III secretion system (T3SS) along with a putative T3SS effector *axoU* (36). Therefore, macrophages could be used as a model system to study *Ax* virulence factors that impact host-pathogen interactions *in vivo*.

Here, a *himar1*-based random transposon mutagenesis screen was performed to identify loci contributing to macrophage cytotoxicity. As part of this screen we identified a giant RTX adhesin, ArtA, encoded by multiple *Ax* genomes. Our results suggest that ArtA promotes *Ax* binding and entry into host cells, a critical step in the cytotoxic phenotype. ArtA is released into supernatants of growing cultures and is recognized by serum from a subset of CF patients colonized by *Ax*, suggesting *in vivo* production and recognition by the host immune response. Phagocytic cells play central roles in the innate immune response suggesting that the manipulation of host defenses likely contributes to the establishment of chronic colonization. These data represent an important first step to elucidate mechanisms of *Ax* pathogenesis for the rational design of diagnostics and therapies.

## RESULTS

### Cytotoxicity precedes bacterial proliferation during *Ax*-macrophage infection.

**FIG 1:**
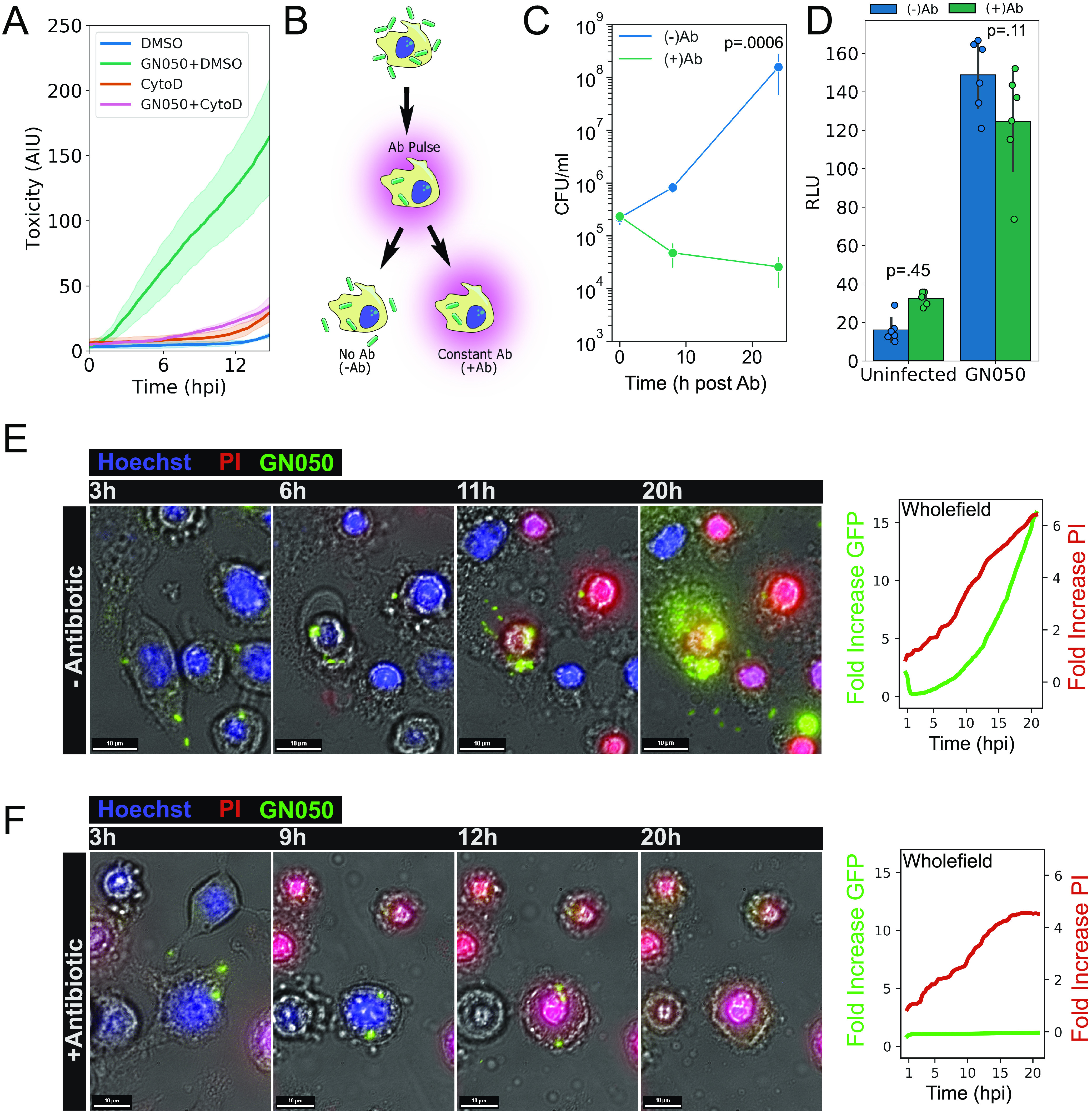
Growth of *Ax* GN050 during macrophage infection. (A) J774a.1 cells were pretreated with cytochalasin D or an equivalent volume of DMSO prior to GN050 infection (MOI: 1). PI was included over the course of infection and was quantified using CellProfiler software as a readout for host cell toxicity. The solid line is the mean, and the shaded areas are the error from four fields of view. (B) Infection schematic for subsequent assays in the figure. J774a.1 cells were incubated with GN050 for 30 min to allow for phagocytosis followed by treatment with gentamicin and polymyxin B to kill extracellular bacteria. Infection medium was replaced with fresh medium (−Ab) to allow intracellular and extracellular bacterial growth or antibiotic containing medium (+Ab) to select for intracellular bacteria only. (C) Quantification of GN050 (MOI: 1) during J774a.1 infection under −Ab or +Ab conditions (*P* = 0.0006 at 24 hpi). Data points are the mean of three independent infections (*n* = 6). (D) Cytotoxicity of GN050-infected J774a.1 cells (MOI: 1) under −Ab or +Ab conditions as determined by AK release (*n* = 6). Data were assessed by two-way ANOVA followed by Tukey’s *post hoc* analysis. (E and F) Live imaging analysis of GN050-infected J774a.1 cells (MOI: 1) from 1 to 20 hpi under −Ab (E) or +Ab (F) conditions. Stills from the live imaging were cropped and merged (left). Quantification of host cytotoxicity (fold increase PI) and GN050 proliferation (fold increase GFP) from full capture fields are on the right. Images and quantification are from a single representative infection that was performed at least on three separate occasions. Scale = 10 μm.

We previously reported that a subset of *Ax* isolates induce cytotoxicity in primary macrophage and macrophage like cell lines, which is dependent on bacterial uptake (36). These observations were confirmed using live imaging of propidium iodide (PI) incorporation into the nuclei of damaged host cells infected with *Ax* strain GN050 (Fig. 1A). The cytotoxic phenotype was abrogated once cytochalasin D, an inhibitor of actin polymerization, was supplemented in the infection (37). To characterize bacterial growth in relation to host cell toxicity the experimental design as outlined in Fig. 1B was utilized. After a brief period of coincubation, a combination of gentamicin and polymyxin B, antibiotics that are impermeable to eukaryotic membranes, was applied to infected cultures to kill extracellular bacteria. Intracellular bacterial populations (+Ab) decreased over the course of 24 h while groups allowing for extracellular replication (–Ab) grew nearly 3 logs. These results suggest that a majority of GN050 replication occurs extracellularly during infection (Fig. 1C). Cytotoxicity was not dependent on bacterial growth. Infections treated with constant antibiotics (+Ab) resulted in similar levels of adenylate kinase (AK) release from lysed cells as those in the absence of antibiotics (Fig. 1D).

In an infection protocol similar to that shown in Fig. 1B, we monitored growth of GN050 that expresses super folding green fluorescent protein (pDBD2*sfGFP*) in relation to host cell toxicity by using live imaging with PI incorporation. Bright field (BF) imaging allowed detailed observations regarding cellular pathology. During infection without antibiotics, bacterial proliferation was preceded by J774a.1 membrane blebbing and PI incorporation in nearly all fields analyzed (Fig. 1E and Movie S1 in supplemental material) and these events were quantified (Fig. 1E and Movie S2). Alternatively, during infection with constant antibiotics (Fig. 1F and Movie S3), where extracellular populations could not replicate, the events of host cell toxicity and bacterial proliferation were distinct. While the PI signal increased to a similar extent as in Fig. 1E, we observed no increase in GFP signal, suggesting that GN050 was not replicating (Fig. 1F and Movie S4). Collectively, these data support a model where GN050 is internalized and survives in the intracellular environment until host cell lysis and bacterial egress. Cellular lysis is followed by bacterial replication, mainly in the extracellular environment.

### Intracellular *Ax* remain in a vacuole prior to host cell death.

**FIG 2:**
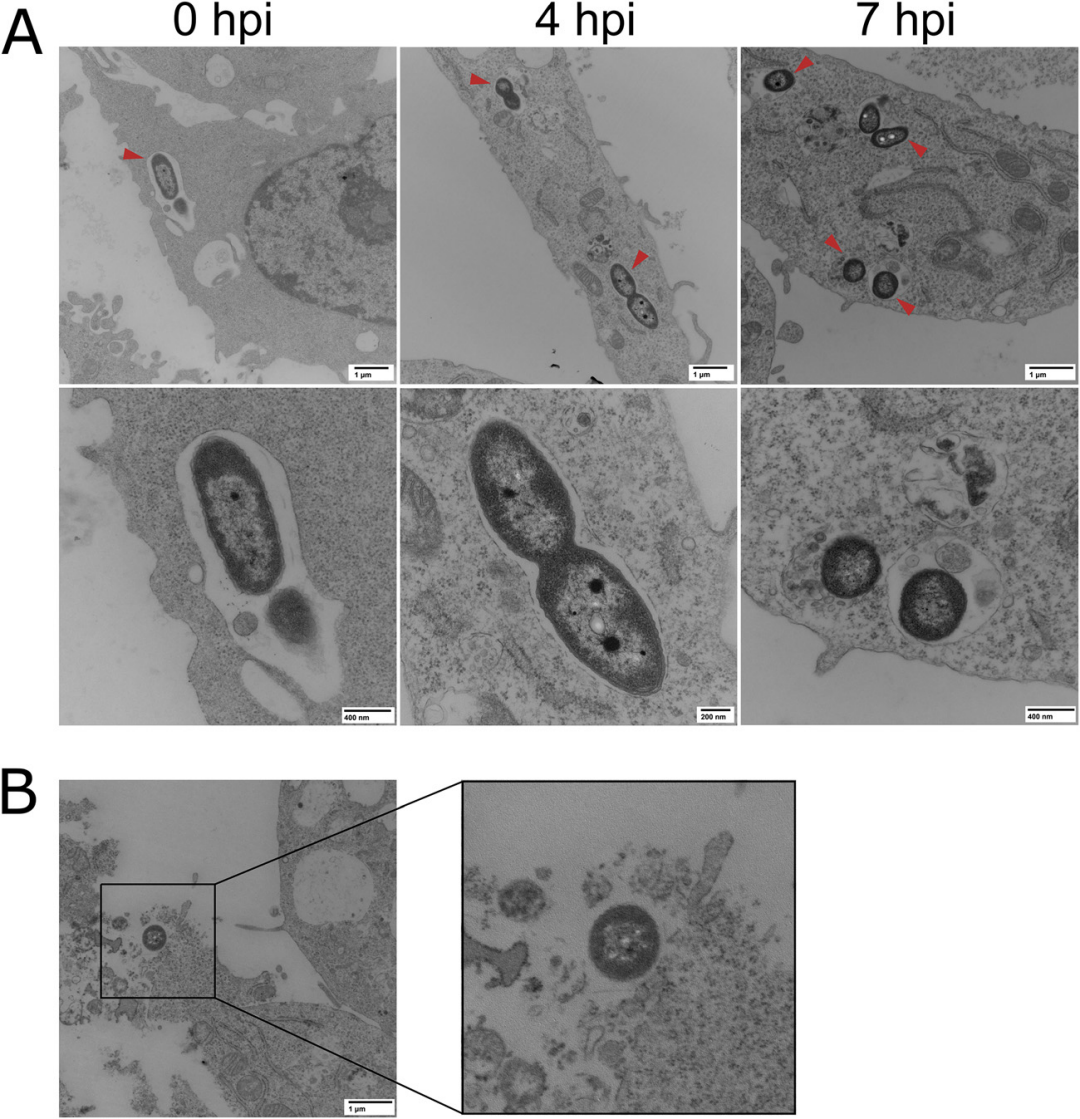
Subcellular localization of *Ax* GN050 during macrophage infection by transmission electron microscopy. (A) J774a.1 macrophages were infected with GN050 (MOI: 1) for 30 min and washed, and the medium was replaced with antibiotic-containing medium for 30 min. Antibiotic medium was replaced with fresh medium and samples were taken at 4 hpi and 7 hpi. An initial time point (0 hpi) was taken at 5 min postinoculation. (A) Vacuole-based GN050 (red arrows) were observed at all time points at low (top) and high (bottom) magnification images. (B) Cellular lysis and bacterial egress from a GN050-infected J774a.1 cell at 7 hpi. Representative images are from two independent infections.

Based on our live imaging data, we estimated that viable GN050 can persist up to 9 h postinternalization before cellular lysis and extracellular bacterial proliferation (Fig. 1E and F). Transmission electron microscopy was used to determine the subcellular location of GN050 prior to host cell lysis. After selecting for intracellular bacteria, a majority of the GN050 cells appear within a membrane-bound vacuole up to 7 h postinfection (hpi; Fig. 2A). Prior to cellular lysis and bacterial egress, vesicles (possibly part of the endocytic pathway) appear to accumulate. Over the course of the infection, vacuoles generally contained one to three bacteria, supporting our previous results that the majority of GN050 replication occurs extracellularly. We conclude that this clinical isolate is able to survive within a vacuole for a sufficient period of time to induce a cytotoxic response, escape, and replicate in the environment.

### Transposon mutagenesis of *Ax* strain GN050.

**FIG 3:**
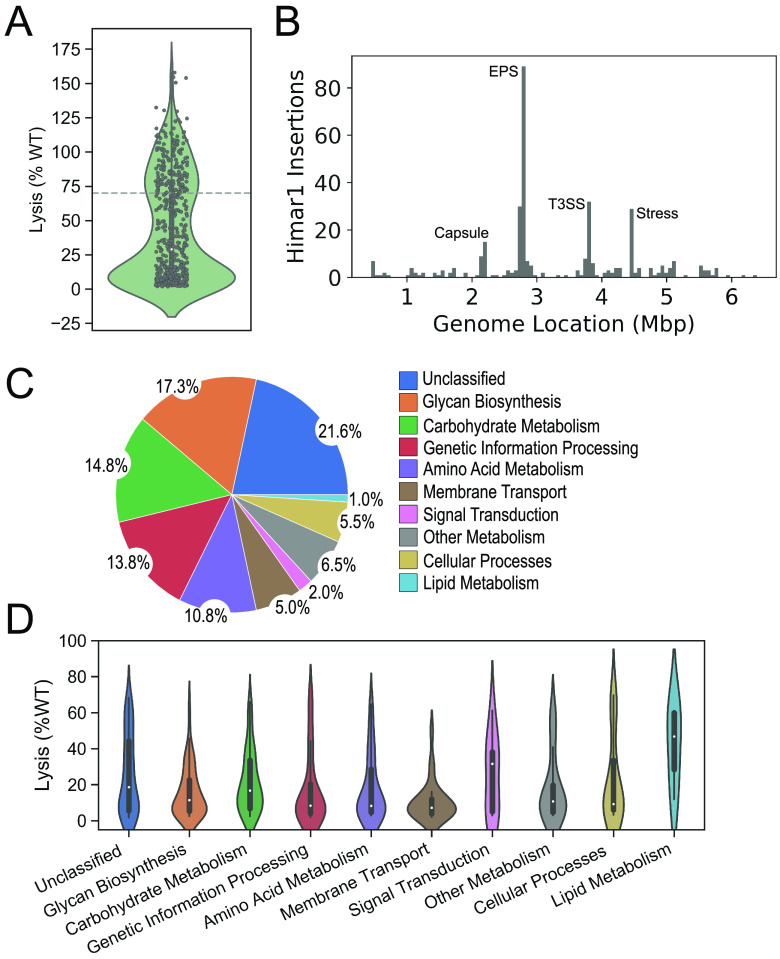
Transposon mutagenesis of *Ax* strain GN050 (A) Cytotoxicity of J774a.1 macrophages infected with GN050 mutants (MOI: 10 for 8 h) identified in the initial CV-based screen was reassessed in a secondary screen using an AK release assay (*n* = 2). GN050 mutants equal to or below 70% of WT cytotoxicity (dashed line) were subjected to further analysis. (B) Identified *himar1* insertions sites across the *Ax* genome when individual insertion events from each mutant were compiled. Each bar represents a 64.5-kb segment of the GN050 genome. (C) Classification of functional pathways involved in macrophage cytotoxicity. Amino acid sequences of ORFs containing *himar1* insertions were annotated using KEGG databases. (D) Data sets from panels A and C were merged to visualize the relationship between a cytotoxicity defect of a mutant and the disrupted pathway.

To identify bacterial genes involved in macrophage cytotoxicity, we performed transposon mutagenesis with a modified *himar1* transposon (Table S1). Individual transposon mutants were suspended in tissue culture medium to deliver a theoretical multiplicity of infection (MOI) of 50 to 200 to J774a.1 macrophages seeded in 96-well plates. Cells were then stained and washed to detect the retention of crystal violet, indicating cell layer integrity and a nontoxic phenotype (38). We screened 18,119 GN050 mutants, and noncytotoxic strains were subcultured onto antibiotic-containing agar medium to confirm the presence of the transposon. This primary screen identified 599 mutants; 525 of these had growth characteristics comparable to the wild-type (WT) strain and were subjected to a secondary screen in which the MOI was standardized to 10. After 8 h of infection, cellular supernatants were assayed for adenylate kinase (AK) release to quantify cellular lysis. Mutants that induced release of less than 70% of the AK released by the parent strain GN050 were considered defective for cytotoxicity and retained to map the transposon insertion site. Of the 525 mutants tested, AK release induced by 368 mutants fell at or below the 70% criterium. Infection by a majority of the mutants resulted in AK release less than 25% that of the parental level (Fig. 3A).

To map the genomic location of each transposon insertion, genomic DNA (gDNA) was purified and digested with a restriction enzyme that does not recognize sequences within the *himar1* transposon. gDNA was ligated and transformed into DH5α *λpir* and selected on medium with 30 μg/mL of chloramphenicol. The resulting plasmids were purified and flanking genomic regions sequenced using outward reading primers that hybridize at each end of the transposon (Supplemental Methods). With the use of this method, 354 of the 368 identified mutants were rescued and sequenced. The remaining 14 mutants displayed poor growth patterns or sequence analyses failed after multiple attempts. Sequenced insertion sites were mapped back to the GN050 genome (Fig. 3B). From the 354 rescued plasmids, we mapped 313 unique *himar1* insertion sites in 163 open reading frames (ORFs) and 27 intergenic regions.

### Classification of pathways involved in macrophage cytotoxicity.

To identify major bacterial pathways involved in host cell cytotoxicity, mapped genes were functionally annotated against Cluster of Orthologous Genes (COG) and NCBI Prokaryotic Genome Annotation Pipeline (PGAP) databases. When possible, amino acid (aa) sequences were categorized as functional pathways using Kyoto Encyclopedia of Genes and Genomes (KEGG) databases (Fig. 3C and Table S2). Putative loci with the highest frequency of insertions (Fig. 3B) included genes hypothesized to be involved in Vi capsular biosynthesis, extracellular polysaccharide (EPS) biosynthesis, T3SS, and stress response (Table S2). These data are consistent with a previous study demonstrating transcriptional activation of T3SS genes upon internalization by macrophages (36).

Bacterial metabolic pathways may have variable contributions to host toxicity. To determine if the identified functional pathways displayed diverse cytotoxic phenotypes when disrupted, data sets corresponding to Fig. 3A and C were merged (Fig. 3D). This analysis suggests that disruption of genes involved in membrane transport (i.e., bacterial secretion systems and ABC transporters), glycan biosynthesis, carbohydrate and amino acid metabolism, and genetic information processing have the greatest impact on host toxicity.

### Identification of *Ax* GN050 mutants deficient for host cell association.

GN050-mediated cytotoxicity is dependent on bacterial entry into host cells (36). Therefore, our mutagenesis screen had the potential to identify mutants that were unable to bind or enter macrophages. A cell association screen using J774a.1 macrophages was optimized and performed. Using our annotated genes from the transposon insertion list (Table S2), we selected mutants with disrupted genes that were putatively involved with biosynthesis or transport of surface-exposed substrates, hypothesizing that these genes would impact bacterial binding or uptake (Table 1). Collectively, the selected loci contained multiple unique gene insertions, and to minimize polar effects, ORFs with insertions that were located at the end of a putative operon were selected. To minimize transcriptional readthrough from a strong *Ax* promoter sequence cloned within *himar1* for downstream genes in an operon, mutants containing an insertion in a reading frame opposite that of the ORF were chosen.

**TABLE 1:** Mutants screened for cell association defects

GN050 mutant	%WT cytotoxicity	Gene locus tag	Function
50C9	5.1	HPS44_04570	tadC: tight adherence protein
110G9	22.7	HPS44_23140	tadB: tight adherence protein B
18E8	32.4	HPS44_23230	Ca^2+^-binding protein, RTX toxin related
163A10	2.2	HPS44_11545	Preprotein translocase subunit SecD
112F4	7.0	HPS44_19580	secG: preprotein translocase subunit SecG
187F11	11.8	HPS44_17100	yscD, sctD, ssaD: type III secretion protein D
161D4	5.6	HPS44_09330	cheB: two-component system, chemotaxis family
156D10	7.5	HPS44_25555	fliS: flagellar secretion chaperone FliS
76H9	6.5	HPS44_09825	glycosyltransferase family 4 protein
185E1	59.9	HPS44_24040	fatty-acyl-CoA synthase
141B2	2.9	HPS44_12480	lipB: lipoyl(octanoyl) transferase
85F5	7.7	HPS44_12615	glycosyltransferase
147B5	6.6	HPS44_12630	galE, GALE: UDP-glucose 4-epimerase
32E7	15.3	HPS44_12750	rfbC, rmlC: dTDP-4-dehydrorhamnose 3,5-epimerase

**FIG 4:**
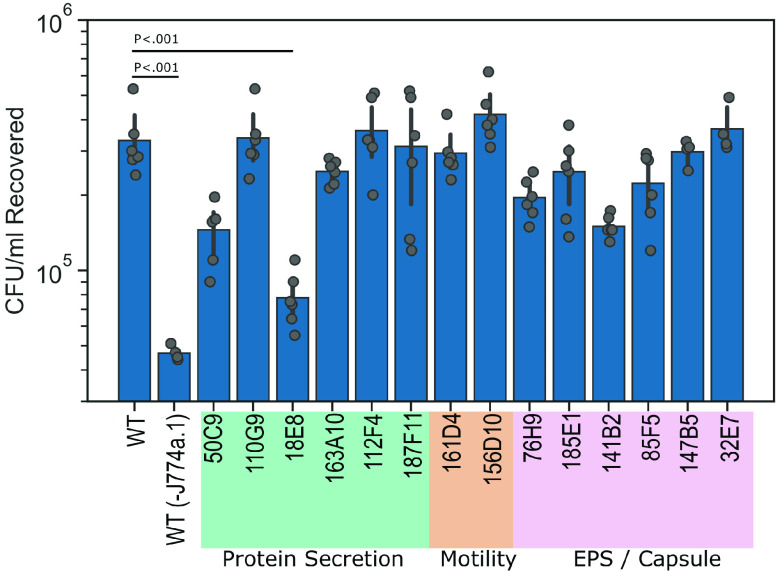
Cell association screen used to identify GN050 mutants defective for host cell binding or entry. GN050 mutant candidates were selected based on disruption of genes predicted to be involved in the production or transport of extracellular substrates. Mutants were incubated with J774a.1 macrophages (MOI: 1) for 30 min followed by two washes to remove extracellular, unbound bacteria. Wells were emulsified and the remaining bacteria were enumerated. To control for background adherence, WT GN050 were incubated without J774a.1 macrophages, WT(-J774a.1). Data bars are the mean, and the error is ± 1 SD. Data points of *n* = 4 to 6 from 2 to 3 biological replicates were assessed by one-way ANOVA followed by Tukey’s *post hoc* analysis.

In total, 14 mutants were selected to screen for their ability to associate with J774a.1 macrophages (Table 1). As a control, WT GN050 was included in the presence or absence of macrophages. The latter allowed us to determine the background of bacteria binding to the tissue culture-treated wells. After coincubation and subsequent washes to eliminate unbound bacteria, the contents of each well were harvested and serially diluted to quantify CFU per milliliters recovered (Fig. 4). We interpret these results as a direct readout for the ability of each mutant to bind and enter host cells. We identified one mutant, GN050 18E8, that was significantly deficient for host cell association (*P* < 0.001) compared to WT GN050 (Fig. 4). Moreover, there was minimal difference between the 18E8 mutant and our background control of WT binding to plastic.

### Genomic characterization of the *Ax* RTX adhesin.

**FIG 5:**
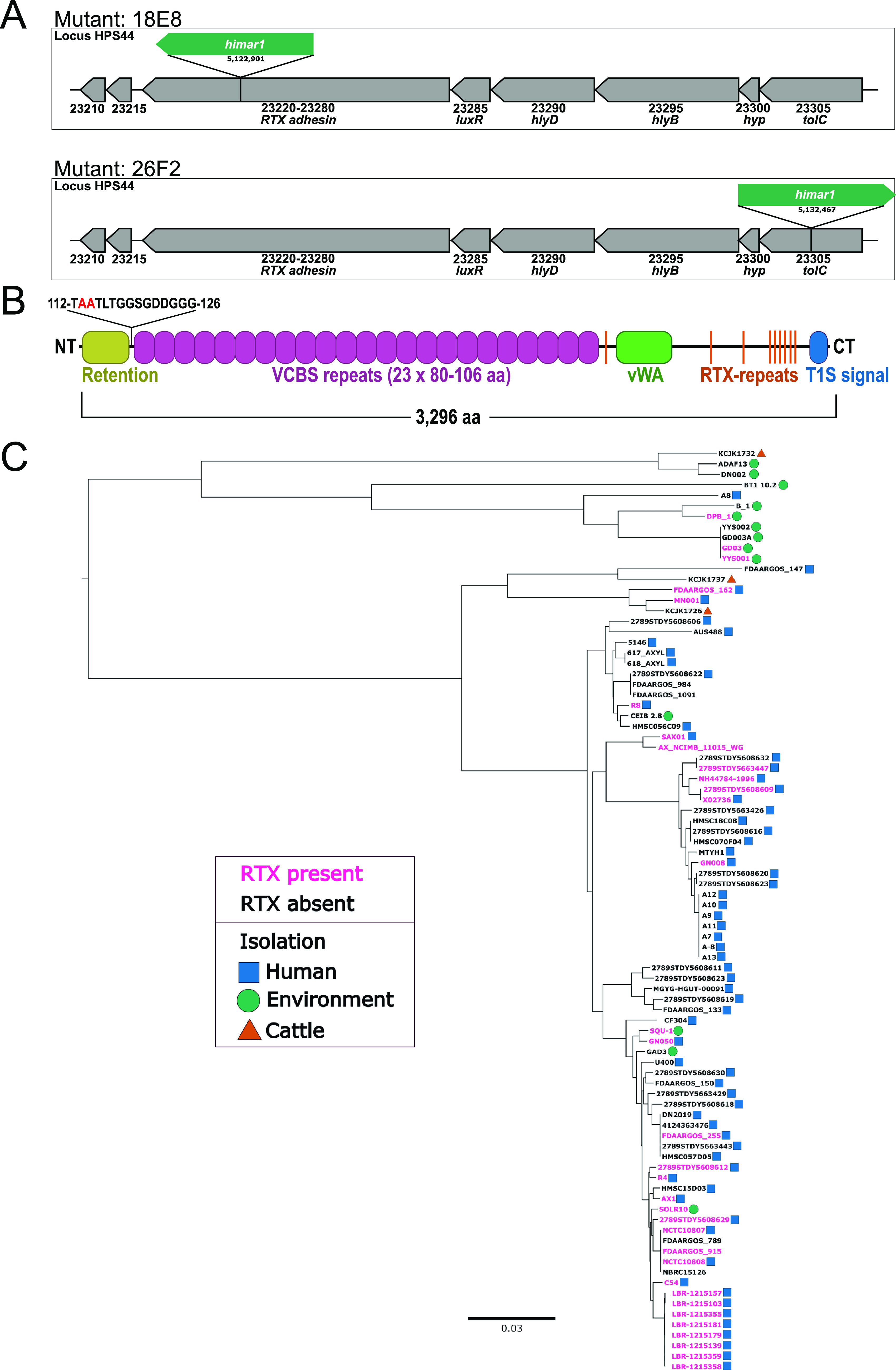
Genomic analysis of the *T1SS*/*artA* locus. (A) *T1SS*/*artA* coding region and location of the transposon insertion in mutants 18E8 and 26F2. Locus tag HPS44_23305, *tolC* OM protein. 23300, hypothetical. 23295, *hlyB* permease/ATPase. 23290, *hlyD* periplasmic adaptor. 23285, *luxR* transcriptional regulator. 23220-23280, *artA* RTX adhesin. 23215, transglutaminase. 23210, EAL domain-containing protein. (B) Domain map of the RTX aa sequence determined by InterProScan. Putative N-terminal cleavage site is highlighted in red. (C) Maximum likelihood phylogenetic tree (midpoint root) of 88 *Ax* isolates based on 77 single-copy core genes. Presence (pink) or absence (black) of full or partial ArtA in *Ax* genomes along with isolation source is denoted. Blue squares, human isolation. Green circles, environmental isolation. Orange triangles, cattle isolation.

The transposon insertion site of GN050::18E8 was mapped to a series of pseudogenes (HPS44_23220-HPS44_32380) originally annotated as an RTX (Repeats In Toxin) toxin (HPS44_23230). Recent updates to the genomic annotation of GN050 confirmed our analysis of the genomic sequence and revealed a hypothetical protein of 3,296 aa annotated as HPS44_RS29835, a retention module containing protein (Fig. 5A and B). In-frame domains were analyzed for their functional significance by using InterProScan (39) (Fig. 5B). The N terminus (NT) encodes a putative cell anchor domain followed by 23 VCBS (*Vibrio*, *Colwellia*, *Bradyrhizobium*, and *Shewanella*) repeating units each ranging from 80 to 106 aa. The C terminus (CT) contains a von Willebrand factor type A (vWA) domain, nine Ca^2+^-binding RTX repeats with the consensus sequence G-G-X-G-(N/D)-D-X-(L/I/F)-X, and a T1SS signal sequence (T1S signal). vWA domains are distributed widely among eukaryotic (40–42) and prokaryotic proteins including pilins (43, 44) and RTX adhesins (45, 46) and often mediate protein-protein interactions. Based on the domain architecture and the absence of an annotated catalytic domain, we conclude that the hypothetical open reading frame likely encodes a large RTX adhesin rather than an RTX toxin, which we will refer to as ArtA (*Achromobacter* RTX Adhesin). Consistent with the presence of a T1SS signal sequence in the RTX adhesin, there was a complete T1SS locus encoding *hlyD* (periplasmic adaptor), *hlyB* (permease), and *tolC* (outer membrane pore) directly upstream of the *artA* locus (Fig. 5A). By expanding the potential number of genes involved in synthesis and secretion, we identified GN050::26F2, which contains a *himar1* insertion in *tolC*, of the postulated T1SS (Fig. 5A). Real-time quantitative PCR (RT-qPCR) analysis revealed that the *T1SS/artA* locus is expressed under normal growth conditions and that mutations in 18E8 and 26F2 lead to a disruption of expression compared to WT (Fig. S1). Overall, we postulate that *artA* is likely expressed in *Ax* as part of an operon containing a complete T1SS for deployment.

We next determined the prevalence of *artA* in 80-eight sequenced *Ax* genomes using an anvi’o workflow (47, 48). Approximately 37.5% (33 of 88) of the genomes in our analysis encoded *artA* (Fig. S2), all with highly similar CT aa sequences (Fig. S3). Due to the poor ORF calls and annotation, this is likely an underrepresentation. A phylogenetic analysis revealed that there was not a coincidence of *artA* presence with strain relationship (Fig. 5C), and a pangenomic analysis showed no major gene clusters uniquely present in *artA* encoding strains (Fig. S4). The presence of *artA* could not accurately be associated with strain isolation source due to the skewed prevalence of human isolates. However, we found that environmental isolates were phylogenetically distinct from human isolates (Fig. 5C), an observation that has been made previously (25). These genomic analyses indicate that a subset of *Ax* isolates encode *artA* identified in our initial screens but that they do not cluster phylogenetically.

### *artA* contributes to host cytotoxicity by promoting bacterial binding and internalization to macrophages.

**FIG 6:**
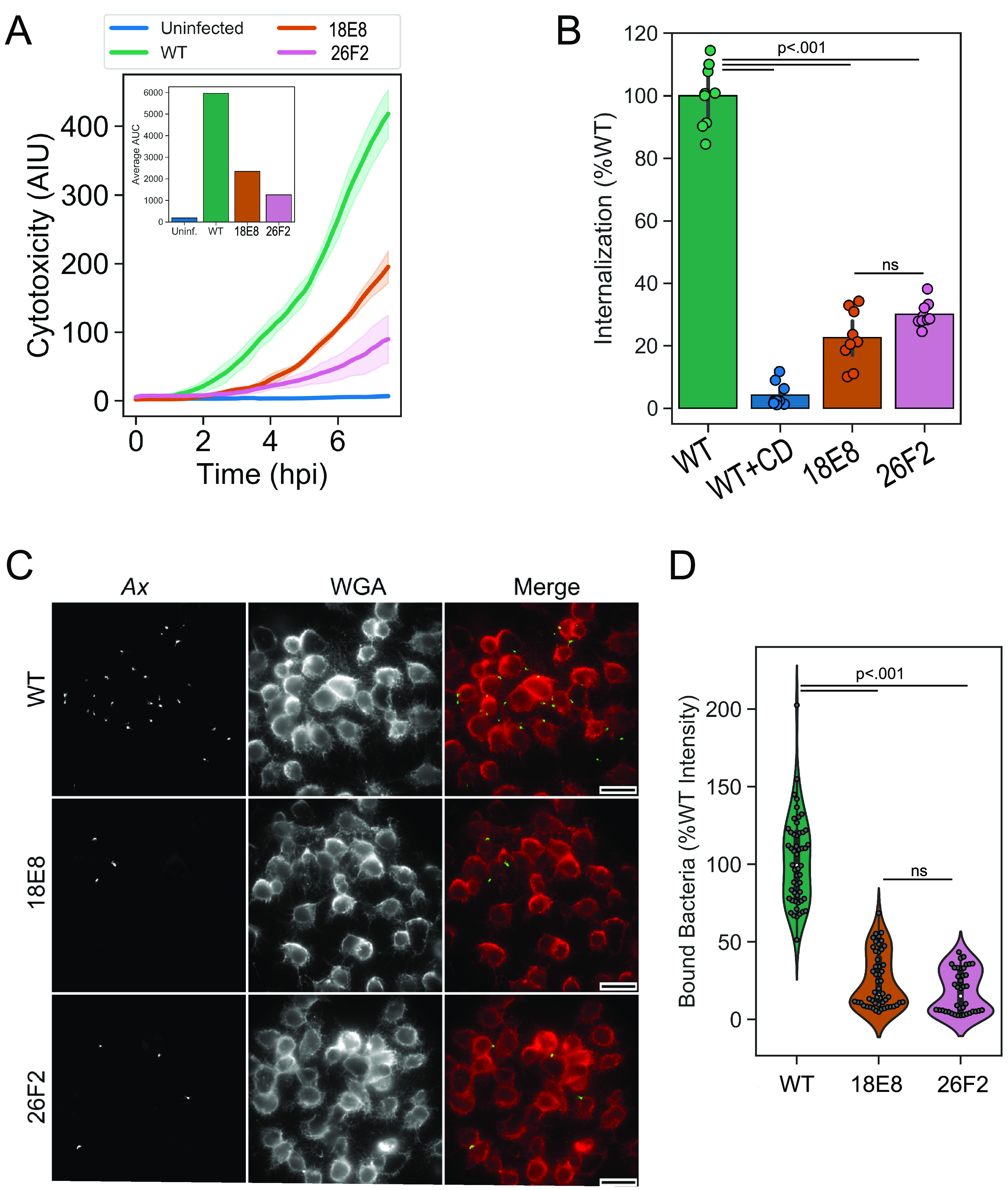
Binding and internalization defects contribute to cytotoxicity defects in 18E8 and 26F2. (A) Live imaging of J774a.1 infected with GN050 or derived strains (MOI: 5) for 8 h. Solid lines are the mean intensity of PI incorporation, or arbitrary intensity units (AIU), into damaged cells from *n* = 4 fields of view (10× objective) and the shaded region is ± 1 SD. The inset is the average area under the curve (AUC) for each group. (B) Internalization of GN050 and derived strains (MOI: 1) after 30 min of phagocytosis by J774a.1 cells. Cells were either pretreated with cytochalasin D (CD) or an equal volume of DMSO. Internalization was normalized relative to WT over the course of three independent experiments (*n* = 9). Data bars are the mean, and the error is ± 1 SD (C) Representative IF microscopy images of J774a.1 cells incubated with WT GN050 or derived strains (MOI: 100). Cells were fixed and labeled with polyclonal antibody recognizing GN050 outer membrane proteins (*Ax*) and wheat germ agglutinin (WGA) to visualize the macrophage membrane. Scale = 25 μm. (D) Quantification of *Ax* signal intensity from 40 to 60 fields of view (10× objective) over 2 to 3 independent experiments and values were normalized to WT binding levels. Data were assessed by one-way ANOVA followed by Tukey’s *post hoc* analysis.

RTX adhesins containing a CT vWA domain are involved in pathogenesis by facilitating cellular invasion (45), infection maintenance (49, 50), and biofilm formation (51). We sought to determine the contribution of *artA* toward macrophage cytotoxicity in *Ax*. Live imaging cytotoxicity curves confirmed the cytotoxic defect of 18E8 and 26F2 (Fig. 6A). The average area under the curve of each infection progression line shows that compared to WT, 18E8, and 26F2 PI staining of host cells was reduced 3-fold or greater over the course of 8 h (Fig. 6A, inset). As 18E8 possesses a defect in host cell association (Fig. 4) and bacterial internalization is required for host cell toxicity (Fig. 1A), we reasoned that the mechanism limiting cytotoxicity by 18E8 and 26F2 may involve bacterial uptake. We measured a 3-fold decrease in bacterial internalization (Fig. 6B) of 18E8 and 26F2 compared to WT and a similar reduction in bacterial binding to host cells (Fig. 6C and D). Importantly, the defect in binding and internalization exhibited by 18E8 and 26F2 appears comparable to the defect in cytotoxicity, which was nearly 30% of WT values in each assay. These data support the model where the*T1SS/artA* locus of GN050 contributes to bacterial binding of the host cell, resulting in bacterial uptake and subsequent cytotoxicity.

### ArtA is detectable in cultured supernatants and is recognized by antibodies in CF serum.

**FIG 7:**
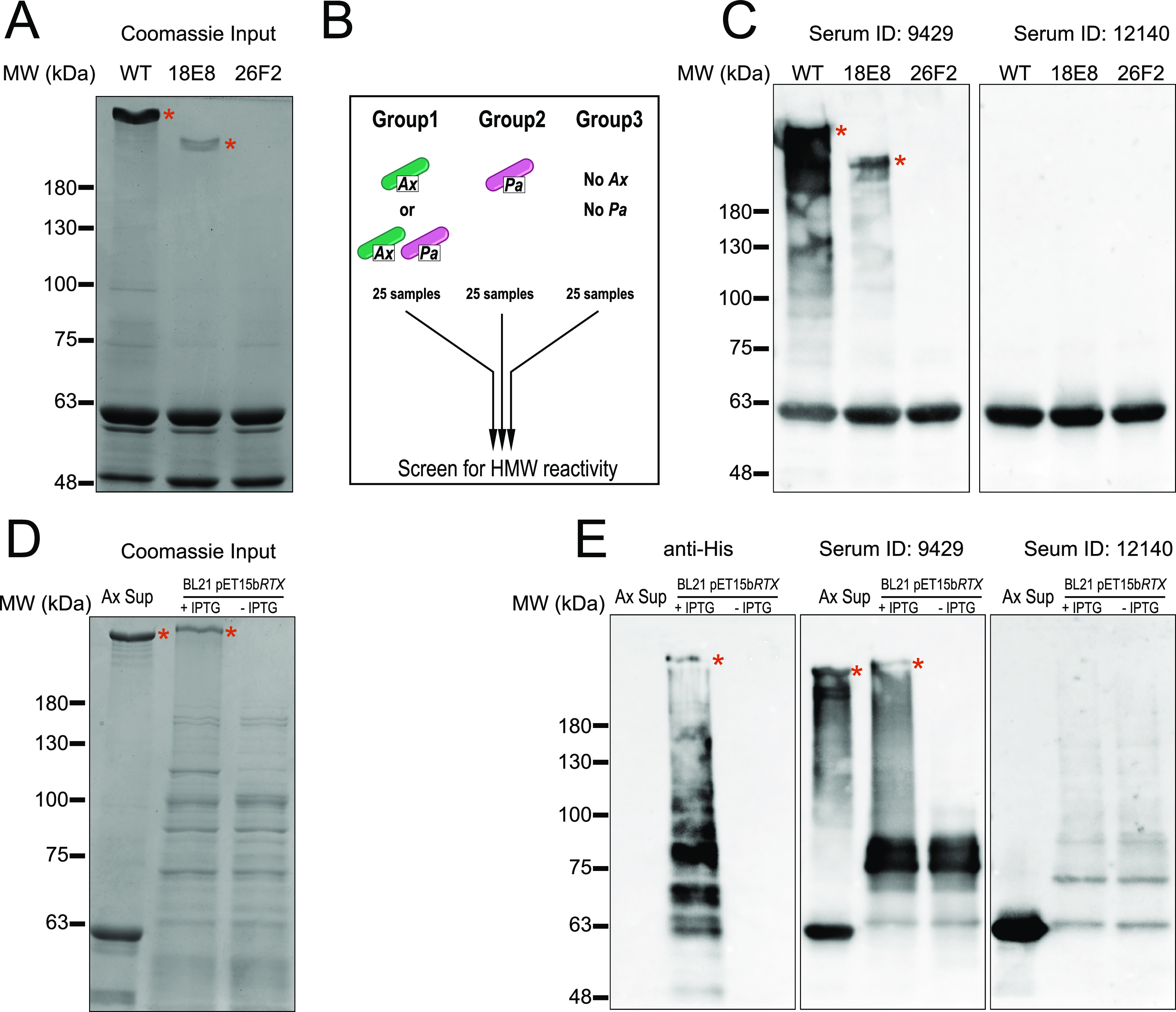
ArtA is released into cultured supernatants and serum from CF patients contain antibodies recognizing ArtA. (A) Coomassie stained SDS-PAGE gel of concentrated culture supernatants from WT GN050, 18E8 and 26F2. (B) Schematic groups of CF serum used to screen for reactivity against ArtA. Group 1, colonization with *Ax* or *Ax* and P. aeruginosa (*Pa*). Group 2, colonization with *Pa* but not *Ax*. Group 3, no history of colonization with *Pa* or *Ax*. (C) Reactivity of group one serum from CF patients with the input antigen from panel A. (D) ArtA production in E. coli BL21 visualized by Coomassie after SDS-PAGE. WT GN050 precipitated supernatant was used as a positive control for the presence of the HMW product (lane 1). ArtA production under induced (lane 2) or uninduced (lane 3) conditions were analyzed. (E) Using the antigen input in panel C, immunoblotting using anti-His, serum 9429 or serum 12140 was used to detect reactivity to the RTX adhesin. In panels A to E, the presence of multiple forms of the GN050 RTX adhesin is marked by a red asterisk. Data are representative of similar experiments performed on multiple occasions.

*artA* appears to be genomically linked to a bacterial transglutaminase-like cysteine proteinase (BTLCP; HPS44_23215). BTLCP-linked T1SS adhesins have been shown to be secreted by a two-step process where the BTLCP cleaves at the NT of the adhesin promoting its release from the cell wall (52). The presence of an NT dialanine cleavage motif in ArtA (Fig. 5B) and a predicted BTLCP encoded downstream of *artA* (Fig. 5A) suggested that the putative peptide may be processed and secreted into the extracellular environment. SDS-PAGE from overnight GN050 liquid cultures demonstrated a high-molecular-weight (HMW) protein that was only present in WT supernatants (Fig. 7A). In supernatants from 18E8 and 26F2, the HMW protein was either reduced both in size and intensity or absent, respectively. We concluded that the HMW product was likely secreted/released ArtA (predicted size of 341.4 kDa).

Next, we aimed to determine if ArtA was expressed during human infection. Patient serum from three cohorts was obtained from the CF Foundation and screened for reactivity against *Ax* supernatants (Fig. 7B). We detected strong reactivity against HMW products in two samples from group one (*Ax* positive) but failed to detect similar patterns in cohorts two or three (*Ax* negative), suggesting that the reactivity was specific to colonization with *Ax* (data not shown). Representative blots (Fig. 7C) of HMW reactive (ID:9429; Fig. 7C, left) and nonreactive (ID:12140; Fig. 7C, right panel) sera from group one with the antigen input stained by Coomassie (Fig. 7A) shows the serum reactivity of serum ID:9429 was specific for ArtA as the HMW banding pattern shifted down or was absent in supernatants from mutants 18E8 and 26F2, respectively. Reactivity against an unidentified product near 63 kDa was present in all groups of sera that we screened, indicating that it may be a common epitope expressed across bacterial genera and not specific to *Ax* (Fig. 7C). Strain GN050 is not of CF origin, and we wanted to determine if CF isolates produced a HMW product that was serum reactive. We found that multiple *Ax* isolates from CF patients (Table S1) produce a HMW band that is serum reactive, suggesting ArtA may be a common epitope (Fig. S5).

We next cloned *artA* from GN050 gDNA into an IPTG-inducible expression vector in frame with an NT histidine tag and transformed Escherichia coli strain BL21. ArtA was detected in whole cell lysates only under inducing (+IPTG) conditions with a similar size to ArtA in *Ax* supernatants (Fig. 7D) and was detected using a His-tag specific antibody (Fig. 7E, left). Serum ID:9429 detected ArtA produced by BL21 only under inducing conditions (Fig. 7E, middle), confirming the specificity of the serum to ArtA. The protein identified in GN050 supernatants appears smaller compared to the protein produced in BL21 (Fig. 7D, lanes 1 and 2). This may represent a processed form of ArtA in GN050 supernatants as BL21 does not encode any annotated processing or secretion machinery required for ArtA secretion. Alternatively, the addition of a His-tag may alter the migration of the protein *in vitro*. These data suggest that our cytotoxic strain of *Ax*, GN050, produces and secretes ArtA that was identified in our screen and expression of a recombinant, tagged version was achieved. Reactive serum also recognized HMW products from multiple CF isolates.

## DISCUSSION

**FIG 8:**
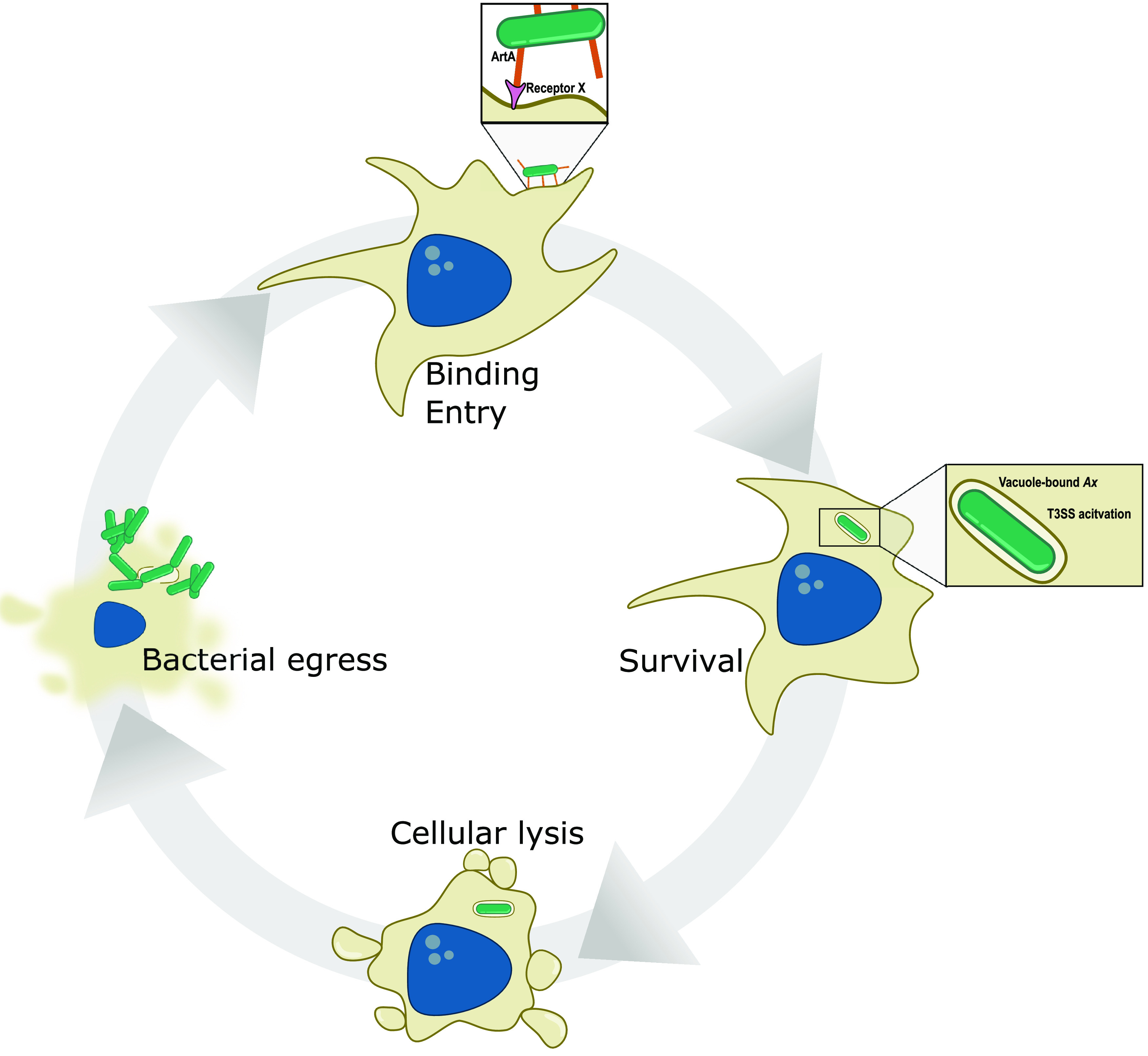
Proposed model for GN050 toxicity in macrophages. The GN050 infection cycle begins by binding to the host cell which is enhanced by the GN050 RTX adhesin ArtA binding to a putative receptor. After bacterial internalization, GN050 remains in a vacuole where T3SS genes and a putative toxin, *axoU*, are transcriptionally activated (36). Survival of vacuole-based GN050 for up to 9 h is followed by host cell membrane blebbing and rupture. GN050 is then released into the extracellular space where growth occurs followed by subsequent internalization events.

The occurrence of *Achromobacter* infections, especially in CF patients, has increased significantly in recent years, yet an understanding of the basic biological processes that contribute to disease is a major knowledge gap. To assess *Ax* pathogenesis, the most prevalent species implicated in human disease, we characterized the relationship of a cytotoxic *Ax* isolate, GN050 (36), with macrophages. GN050 was internalized and able to survive host defenses within a vacuole for up to 9 h, after which host cell lysis occurred allowing the internalized bacteria to replicate in the extracellular environment. Our group has previously reported that this cytotoxic event was dependent on bacterial internalization and correlates with transcription of T3SS genes and a toxin effector, *axoU* (36). We therefore propose the model in Fig. 8 where GN050 persists in a vacuole where it is likely that virulence-related factors are deployed leading to cellular lysis. Using our optimized model, we performed transposon mutagenesis, which allowed us to potentially identify genes involved in bacterial uptake, intracellular survival, and the induction of a cytotoxic response, events leading to host cell lysis.

Our screen identified multiple genes in loci involved in Vi capsular biosynthesis (53), EPS biosynthesis, and an RseA/RseB stress response system responsible for degrading misfolded proteins in the periplasm (54–56). The former two were not involved in bacterial association with host cells (Fig. 4), suggesting they may support bacterial integrity in the host, possess endotoxin activity, or protect the bacterium from endocytic breakdown. In agreement with our previous transcriptional data, 14 unique genes belonging to the T3SS locus were found to mediate host cytotoxicity. It is possible this system secretes effectors from within the *Ax*-containing vacuole to manipulate host defense systems and allow for bacterial persistence or to induce a cytotoxic response. Our screen did not identify individual T3SS effectors, including AxoU, indicating that the contribution of multiple effectors may be needed to induce cellular lysis. While the screen yielded intriguing results, we note that there are limitations to the approach. A majority of the identified genes contained a single disruption, and therefore, our screen did not saturate all possible *himar1* insertions sites within the GN050 genome. Furthermore, transcriptional readthrough of antibiotic resistance genes present on *himar1* has the potential to cause polar effects outside of the gene or locus of interest. It will be therefore essential to assess transcription of genes surrounding a particular *himar1* insertion site using RT-qPCR.

Our efforts focused on identifying loci that promote bacterial association with host cells, an essential first step in the cytotoxic process. This led to the characterization of a giant RTX adhesin, termed *artA*, and a T1SS locus. RTX adhesins are large proteins with an N-terminal membrane anchor, large repetitive extender regions, and a ligand binding CT. At >2,000 aa, they are often the largest proteins produced by bacteria and can be incompletely annotated due to the presence of numerous repetitive modules (57). We utilized an anvi’o pipeline to detect a highly conserved ArtA CT with a vWA domain, RTX Ca^2+^ binding repeats, and a T1S signal sequence (Fig. S3) in nearly 38% of sequenced *Ax* genomes (Fig. 5). vWA domains are present throughout eukaryotic biology including I-domains of integrins, complement factors, and von Willebrand factor (vWF) plasma proteins. These domains facilitate protein interactions and cell-to-cell adhesion by binding to collagen of the extracellular matrix, glycoproteins, and other vWA domain-containing proteins (58–63). RtxA of *L. pneuomophila* also contains a C-terminal vWA domain that promotes bacterial entry into human macrophages, entry into amoebae, and virulence in mice (45, 64, 65), and it is likely that ArtA in GN050 functions similarly. Studies involving insertion mutants of either *artA* or the T1SS delivery machinery concluded that the locus contributes to cytotoxicity in J774a.1 macrophages by promoting binding and entry of GN050 into macrophages (Fig. 6). We were able to perform random mutagenesis in GN050; however, we were unable to complement 18E8 and 26F2 insertion mutants due to the lack of available genetic tools for *Ax*.

By analyzing cultured supernatants, we were able to detect secreted ArtA in the medium. Encoded upstream and transcriptionally independent of *artA* (Fig. S1) is a BTLCP (HPS44_23215), which is often linked with T1SS machinery and large RTX adhesins (66). The BTLCP of Pseudomonas fluorescens, LapG, is a periplasmic cysteine protease that is responsible for cleavage of the giant T1SS RTX adhesin, LapA, at an N-terminal dialanine motif leading to removal of LapA from the surface and reducing biofilm maintenance (52, 67, 68). The presence of a dialanine cleavage motif in ArtA (A113 to A114), along with our biochemical data, suggest that the giant adhesin is processed in a similar manner. Determining the regulation of retention and release of ArtA from the bacterial surface will be important to understand its role in pathogenesis. Furthermore, antibodies in CF serum from patients colonized by *Ax* reacted toward secreted ArtA. This implies that ArtA is produced *in vivo* by a subset of *Ax* isolates, allowing it to be a target of the immune system. We speculate that during colonization *Ax* may utilize ArtA to preferentially associate with macrophages leading to cellular lysis and further damaging of host tissue.

This study identified ArtA, a giant RTX adhesin that promotes cytotoxicity. The receptor for ArtA and the mechanism of regulation are unknown. Importantly, we have characterized a virulence factor in *Ax* when functional studies have lagged due to lack of robust genetic systems and cell models to study pathogenesis. This work shows that our optimized cell model can be used to identify and functionally characterize virulence determinants in *Ax*, building a greater understanding of how this pathogen contributes to disease.

## MATERIALS AND METHODS

### Bacterial culture and tissue culture.

*Ax* GN050 (36) and derived strains were grown on Lysogeny Broth (LB) agar or LB supplemented with 100 μg/mL chloramphenicol for 1 day at 37°C followed by another day at room temperature (RT). J774a.1 mouse macrophage cells (ATCC) were seeded for bacterial infections in Dulbecco’s modified Eagle’s medium (DMEM; Thermo Scientific) supplemented with 10% heat-inactivated fetal bovine serum (FBS; Atlanta Biologicals) a day prior to the assay. Tissue culture cells were maintained at 37°C in humidified air containing 5% CO_2_.

### Bacterial infections.

Excluding the transposon mutagenesis screen (Supplemental Methods), all other bacterial infections were performed in a similar manner. Two-day-old *Ax* cultured on LB agar were emulsified in 900 μL of DMEM. The optical density at 600 nm (OD_600_) was recorded, and 100 μL of the bacterial emulsion was diluted into 1 mL of DMEM. A final dilution into serum-free DMEM was carried out to achieve an MOI of 1 to 10, depending on the assay. Bacterial MOIs were confirmed by CFU after serial dilution. For AK release assays, the reaction was performed as previously described (36). For live imaging of cytotoxicity, reagents were supplemented with Hoechst 33342 (Invitrogen) at 5 μg/mL for 10 min followed by a single wash with Hanks’ balanced salt solution (HBSS; Thermo Scientific) and PI (Sigma) at 0.36 μg/mL constant.

### Cell association screen.

J774a.1 cells were seeded at a density of 1.5 × 10^5^ cells per well in 24-well plates. GN050 WT and derived *himar1* mutants were used to infect the cells at an MOI of 1 after centrifugation at 600 × *g* for 5 min. Cell binding and internalization were allowed to proceed for 30 min at 37°C, 5% CO_2_. Wells were washed 2× with HBSS followed by scraping the entire contents of the well for serial dilutions onto LB agar plates. Recovered CFU were enumerated.

### Bacterial adherence assay.

J774a.1 macrophages were seeded onto 18-mm glass coverslips #1.5 (Warner Instruments) at a density of 3 × 10^5^ cells per well in 12-well plates. Prior to the addition of bacteria, J774a.1 cells were incubated on ice for 30 min. Each well was then washed with cold DMEM. Approximately 350 μL of bacterial culture diluted into cold DMEM was placed onto the J774a.1 cells for a final MOI of 100. Cells were incubated on ice for 30 min while shaking horizontally followed by a 15-min static incubation. Each well was washed twice with cold HBSS to remove unbound bacteria and then fixed using 4% paraformaldehyde in phosphate-buffered saline. For detection by fluorescence microscopy the following steps were taken: block with 3% bovine serum albumin (BSA) in PBS for 1 h at RT, probe for GN050 with anti-GN050 OM polyclonal antibody at 1:150 in 3% BSA for 1 h at RT, wash, followed by anti-rat IgG Alexa Flour (AF)-488 conjugate (Thermo Scientific) at 1:1,500 in 3% BSA for 1 h at RT, probe for J774a.1 membrane (wheat germ agglutinin AF-594 [Thermo Scientific] at 5 μg/mL) and nucleus (Hoechst 33342 at 5 μg/mL) in HBSS for 10 min at 37°C, and mount the slides using ProLong Gold antifade with DAPI (Thermo Scientific) and allow to cure overnight, covered at RT. GN050 signal intensity was quantified using CellProfiler software over 40 to 60 fields of view.

### Data availability.

The molecular and genetic tools developed in this study are available upon request.
