# Supplementary material for: The In Vitro Replication Cycle of Achromobacter xylosoxidans and Identification of Virulence Genes Associated with Cytotoxicity in Macrophages
Source: Microbiol Spectr. 2022 Jul 20;10(4):e02083-22. doi: 10.1128/spectrum.02083-22 (PMC9430717; doi:10.1128/spectrum.02083-22)
Supplement: Supplemental file 1 — Supplemental material. Download spectrum.02083-22-s0001.pdf, PDF file, 4.4 MB [file spectrum.02083-22-s0001.pdf]

SUPPLEMENTAL DATA

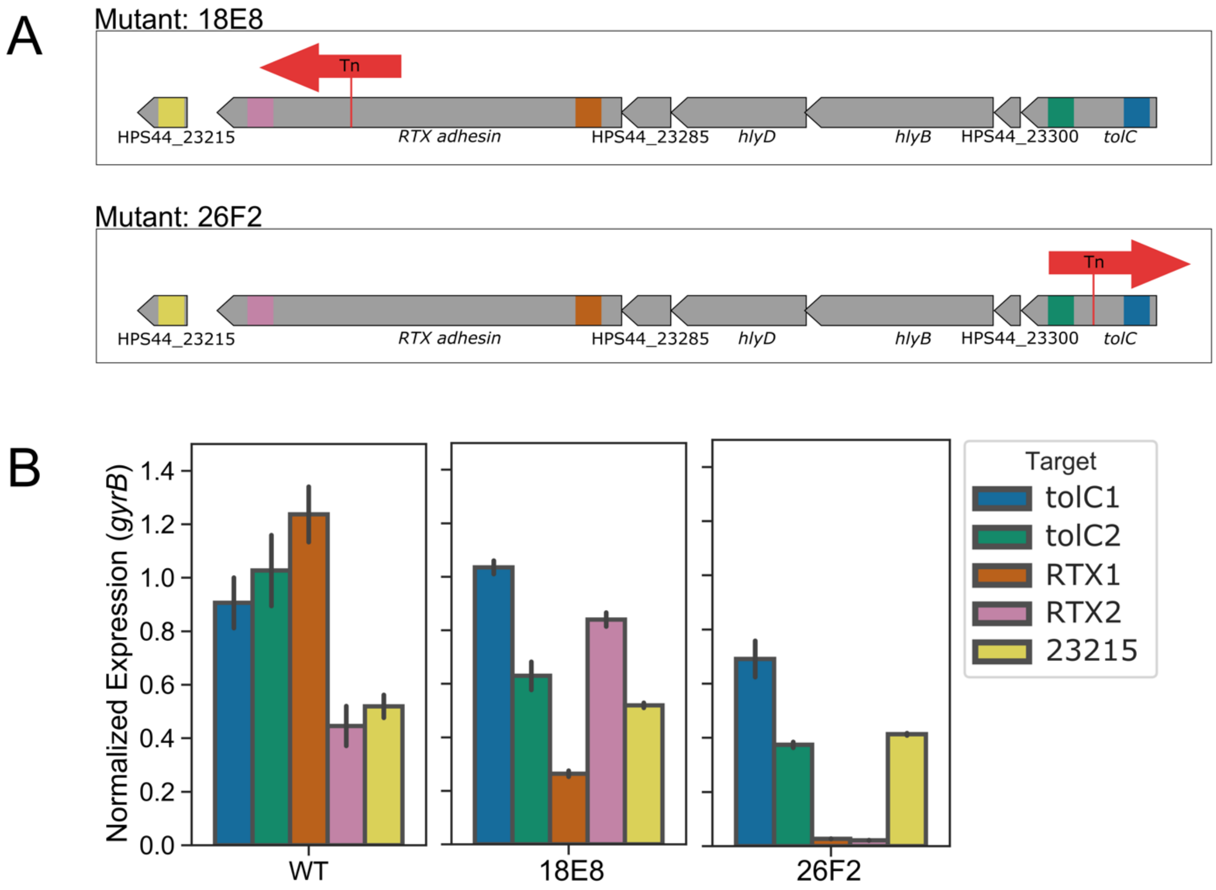

Figure S1. Transcriptional analysis of transposon insertion mutants. (A) Locus map and insertion sites of 18E8 and 26F2 with primer sets in colored boxes used to determine transcript expression. Primers were designed to amplify upstream and downstream of the insertion site in each gene. (B) Expression of target sites in WT GN050, 18E8 (*RTX::Tn*) or 26F2 (*tolC::Tn*) was normalized to expression of the housekeeping gene *gyrB*. Data bars are the mean of 2-3 biological replicates performed in technical triplicate and the error is ± 1 SD.

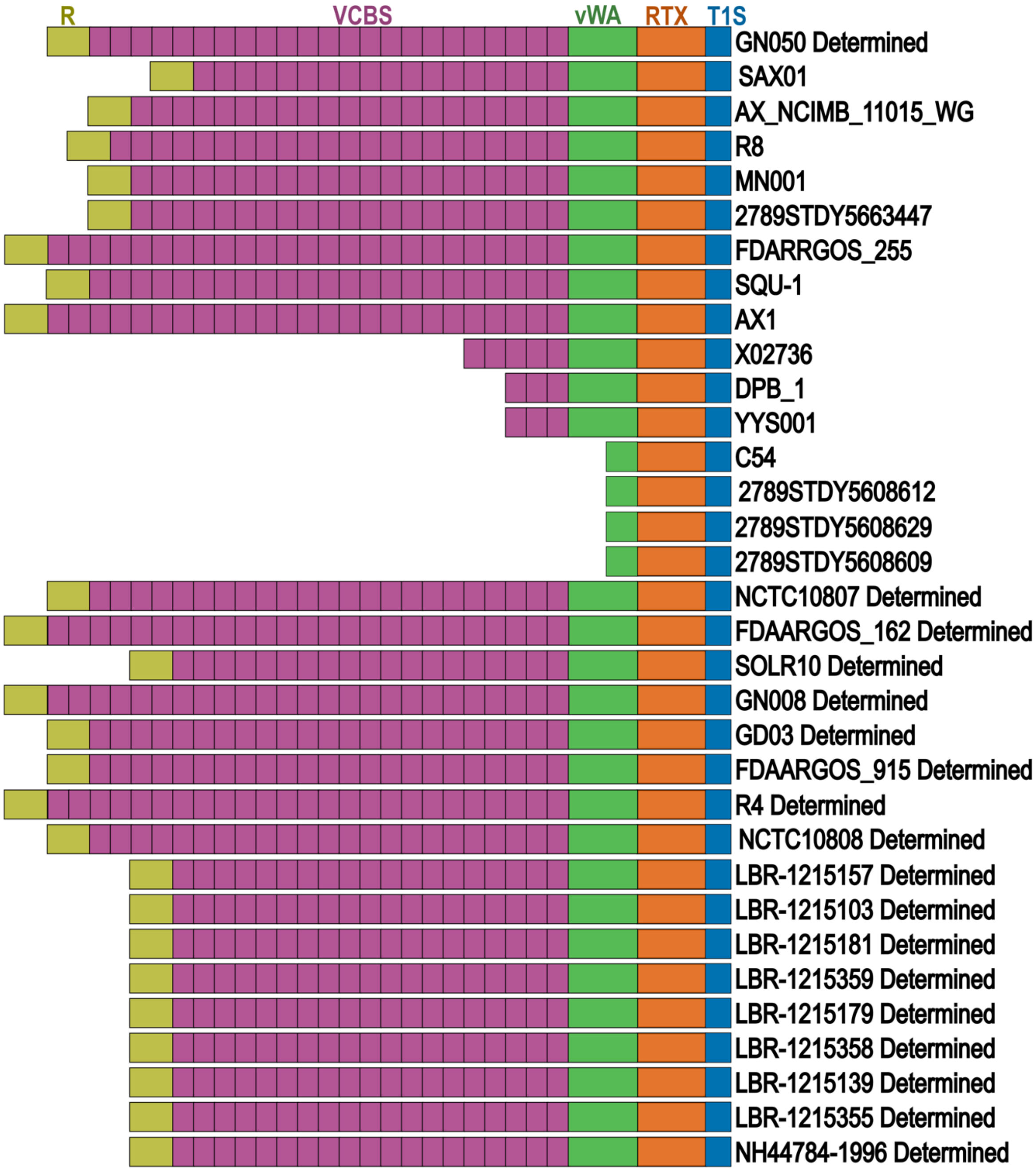

Figure S2. Determined domain architecture of ArtA in 33 *Ax* genomes with respect to the GN050 RTX adhesin. Using an anvi'o pipeline, 8 genomes were found to encode a complete ArtA while 25 other sequences in the gene cluster encode the highly conserved CT including vWA, RTX repeats and T1SS signal sequence. In genomes containing an ArtA CT but lacking further annotation, manual determination of the VCBS repeating units were determined and denoted as "determined." R; retention module. VCBS; repeating units. vWA; von Willebrand type A. RTX; Ca^{2+} binding RTX repeats. T1S; T1SS signal sequence.

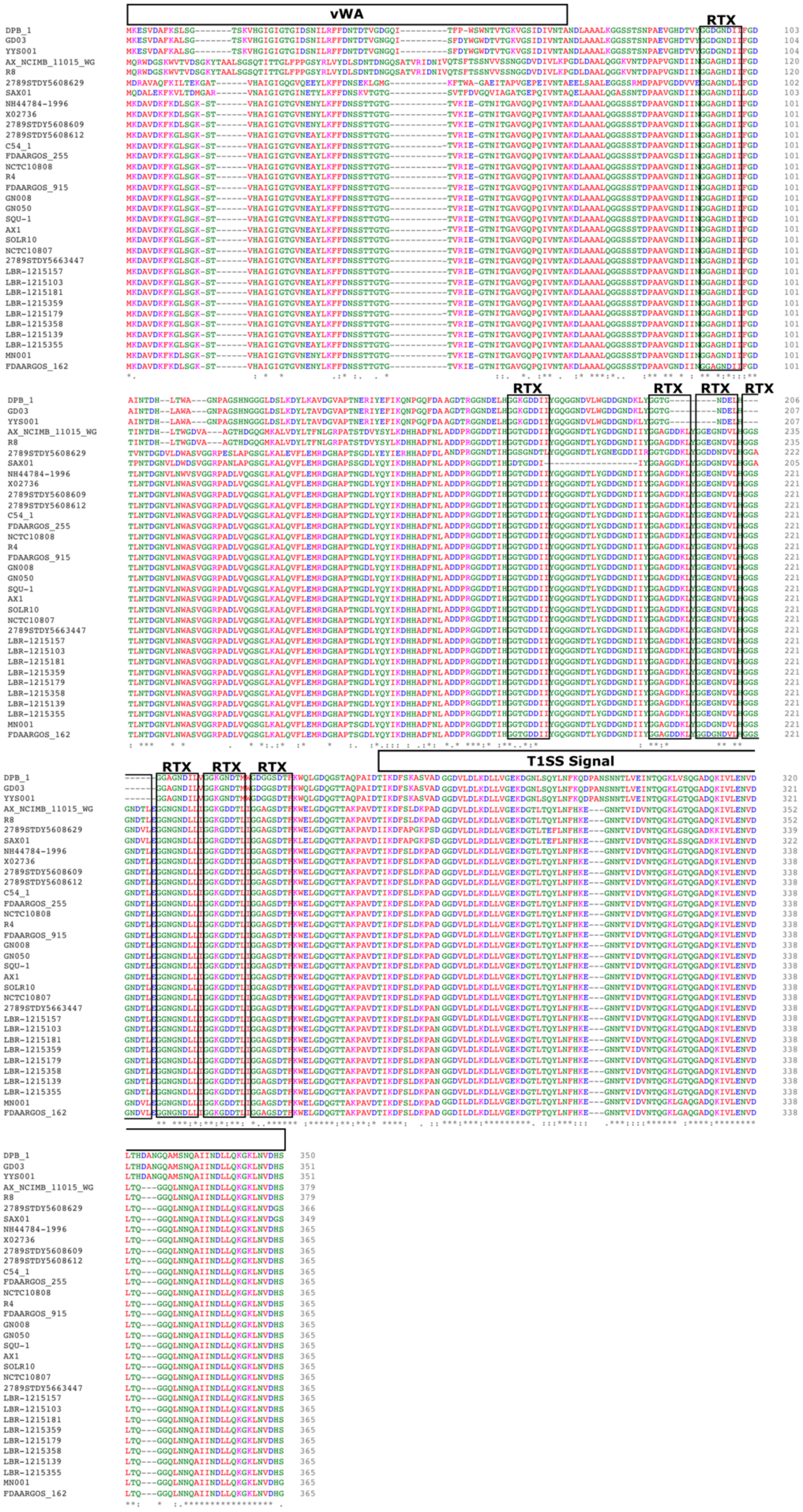

Figure S3. Alignment of CT aa from ArtA in 33 *Ax* genomes. Sequences obtained from the anvi'o analysis and aligned by Clustal Omega. CT domains are annotated.

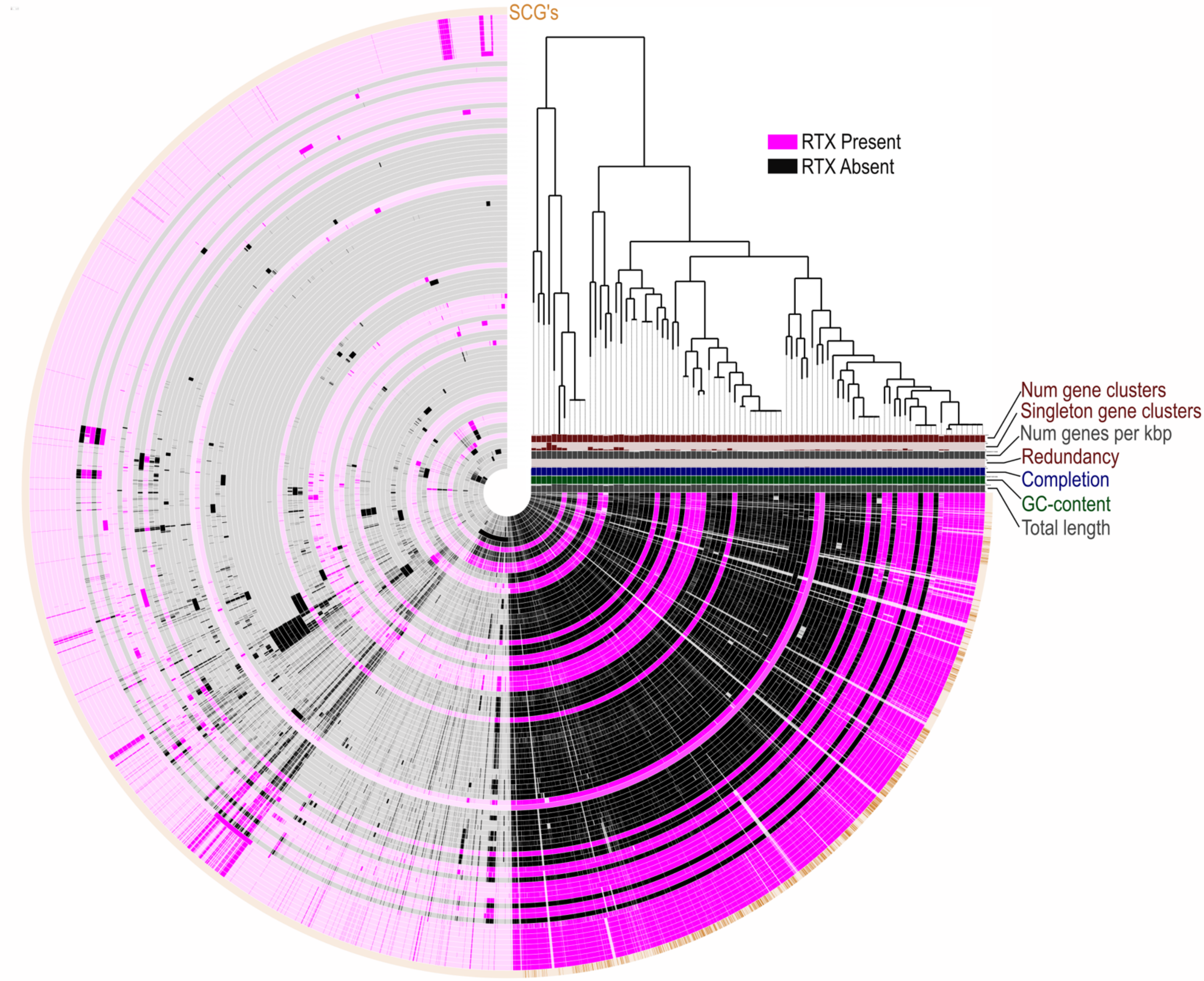

Figure S4. Pangenomic analysis of 88 *Ax* genomes with respect to GN050 synteny. Genome layers are ordered by the maximum likelihood relationship of 77 single-copy core genes. Displayed is the presence or absence of gene clusters of homologous genes based on amino acid sequence identity. By this method, genomes that contained the *artA* gene cluster are shown in pink and genomes that lacked the *artA* gene cluster are shown in black. Minimum requirement for presence of *artA* in a genome included annotation of a vWA domain, RTX-repeats and a T1SS signal sequence (Figure S3).

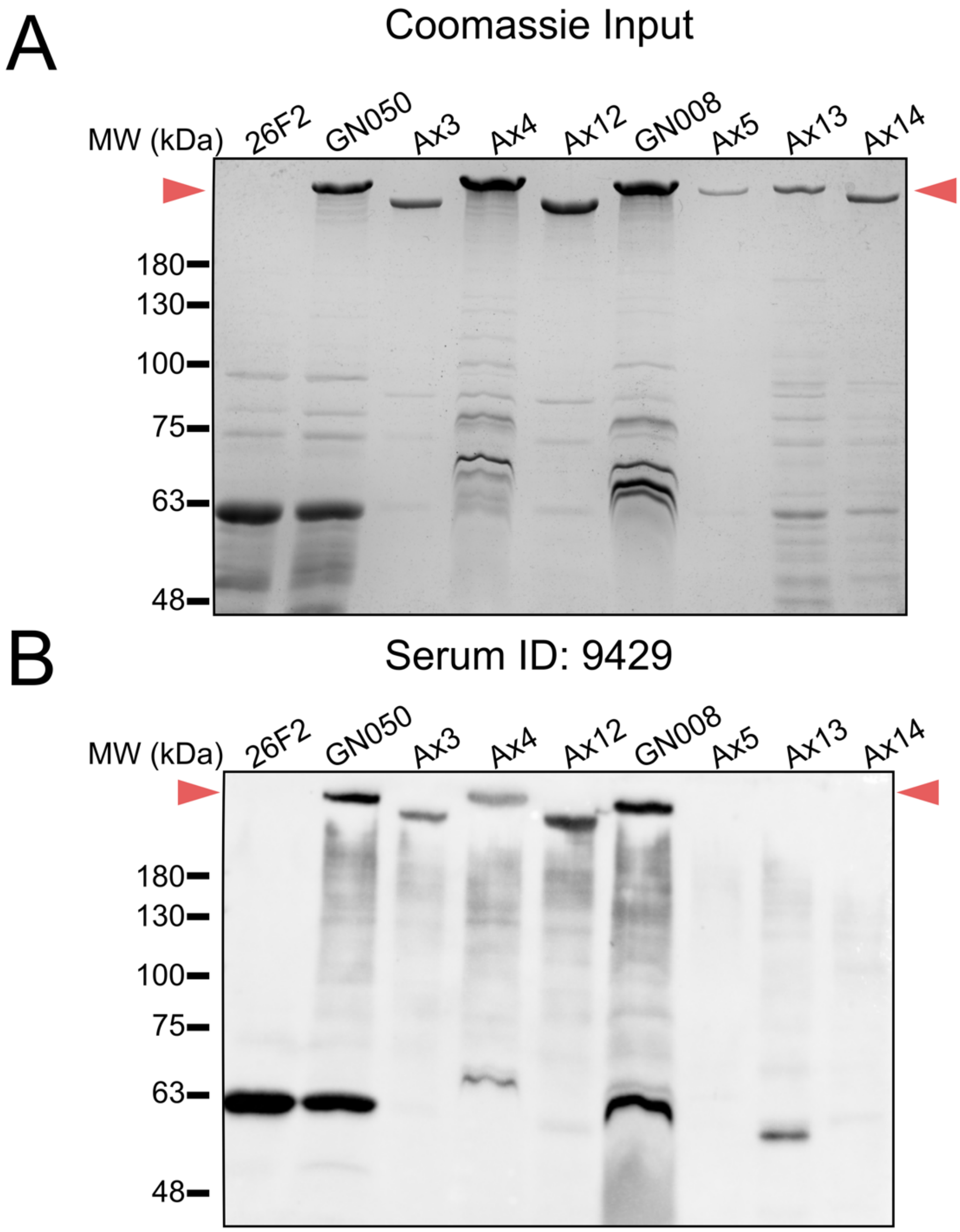

Figure S5. CF serum antibodies that recognize ArtA also recognize a HMW product produced by other *Ax* CF isolates. (A) Precipitated supernatants from overnight cultures of GN050::26F2, WT GN050, or *Ax* CF isolates Ax3, Ax4, Ax12, GN008, Ax5, AX13, AX14. (B) Western blot using CF serum ID: 9429 that was previously found to have GN050 ArtA reactivity. Reactivity of the serum against HMW bands was found in CF isolates Ax3, Ax4, Ax12, and GN008. Detection of a HMW similar to ArtA is denoted by red arrows.

Table S1. Bacterial strains and plasmids used in this study with relevant descriptions.

Strain/ Genotype	Description	Reference
A. xylosoxidans		
GN008	CF isolate	1
GN050	Ear infection isolate	1
18E8	GN050 *artA::himar1*	This study
26F2	GN050 *tolC::himar1*	This study
Ax3	CF isolate	1
Ax4	CF isolate	1
Ax5	CF isolate	1
Ax12	CF isolate	1
Ax13	CF isolate	1
Ax14	CF isolate	1

Plasmid	Description	Reference
pDBD1	Derivative of pCM62 (2)	This study
pDBD2*sfGFP*	pDBD1 with GN050 30S ribosomal protein S21 promoter region and *sfGFP*	This study
Modified *himar1*	30S ribosomal protein S21 promoter region driving *CmΩ* open reading frame added at NheI site of *himar1*	3

Table S2. Compiled list of GN050 *himar1* insertion mutants with relevant information.

Mutant	%WT Toxicity	Location	Gene Locus Tag	Function	Source
59D12	44.9	445,064	HPS44_01820 / HPS44_01825	Intergenic	
156B3	2.9	476,296	HPS44_01970	tyrB; aromatic-amino-acid transaminase	KeggGhostKoala
150G10	4.3	477,901	HPS44_01975 / HPS44_01980	Intergenic	
149H1	9.9	480,415	HPS44_01980	lon; ATP-dependent Lon protease	KeggGhostKoala
85E3	69.7	480,814	HPS44_01985	clpX, CLPX; ATP-dependent Clp protease ATP-binding subunit ClpX	KeggGhostKoala
61F6	37.1	481,968	HPS44_01985 / HPS44_01990	Intergenic	
28D5	63.6	495,682	HPS44_02085	carbohydrate isomerase	NCBI_PGAP
16H10	67.8	547,106	HPS44_02295	putative transposase	KeggGhostKoala
162C10	43.7	583,825	HPS44_02455 / HPS44_02460	Intergenic	
180B7	38.5	635,186	HPS44_02670	hpaI, hpcH; 4-hydroxy-2-oxoheptanedioate aldolase	KeggGhostKoala
41A7	59.0	639,490	HPS44_02690	squalene synthase HpnC	NCBI_PGAP
116G8	66.4	702,028	HPS44_03015 / HPS44_03020	Intergenic	
24B11	58.2	1,031,493	HPS44_04410	tktA, tktB; transketolase	KeggGhostKoala
116E9	2.9	1,065,563	HPS44_04550	hypothetical protein	NCBI_PGAP
50C9	5.1	1,068,113	HPS44_04570	tadC; tight adherence protein	KeggGhostKoala
25B5	3.3	1,070,374	HPS44_04575	cpaF, tadA; pilus assembly protein CpaF	KeggGhostKoala
46A7	5.7	1,079,434	HPS44_04620 / HPS44_04625	Intergenic	
125F10	2.8	1,105,359	HPS44_04720	etk-wzc; tyrosine-protein kinase Etk/Wzc	KeggGhostKoala
162D8	9.4	1,145,736	HPS44_04875	vcaM; ATP-binding cassette, subfamily B, multidrug efflux pump	KeggGhostKoala
113D2	44.3	1,159,820	HPS44_04955	short chain dehydrogenase	NCBI_PGAP
29H7	64.1	1,264,383	HPS44_05495 / HPS44_05500	Intergenic	
115E2	61.3	1,276,873	HPS44_05570	dapA; 4-hydroxy-tetrahydrodipicolinate synthase	KeggGhostKoala
12H7	24.0	1,417,632	HPS44_06255 / HPS44_06260	Intergenic	
13E2	25.0	1,417,632	HPS44_06255 / HPS44_06260	Intergenic	
154E8	7.8	1,504,306	HPS44_06660	aldolase	NCBI_PGAP
155E10	19.7	1,514,265	HPS44_06705	aarC, cat1; succinyl-CoA:acetate CoA-transferase	KeggGhostKoala
30A6	64.7	1,531,250	HPS44_06795	CBS domain-containing protein	NCBI_PGAP
57F6	11.9	1,546,665	HPS44_06875	hypothetical protein	KeggGhostKoala
30C12	5.0	1,557,551	HPS44_06935	pstS; phosphate transport system substrate-binding protein	KeggGhostKoala
196E1	4.6	1,655,395	HPS44_07355	purK; 5-(carboxyamino)imidazole ribonucleotide synthase	KeggGhostKoala
106F7	4.1	1,657,324	HPS44_07375	purC; phosphoribosylaminoimidazole-succinocarboxamide synthase	KeggGhostKoala

101F2	49.2	1,717,670	HPS44_07680	ubiD; 4-hydroxy-3-polyprenylbenzoate decarboxylase	KeggGhostKoala
162B10	10.5	1,735,318	HPS44_07780 / HPS44_07785	Intergenic	
81G4	56.3	1,738,305	HPS44_07785 / HPS44_07790	Intergenic	
29E9	2.4	1,738,366	HPS44_07790	type II toxin-antitoxin system RelE/ParE family toxin	NCBI_PGAP
150G8	33.6	1,875,291	HPS44_08475	hypothetical protein	NCBI_PGAP
167E8	64.6	1,930,755	HPS44_08755	porin	NCBI_PGAP
161D4	5.6	2,062,402	HPS44_09330	cheB; two-component system, chemotaxis family, protein-glutamate methylesterase/glutaminase	KeggGhostKoala
104H9	66.8	2,114,136	HPS44_09600	rsmD; 16S rRNA (guanine966-N2)-methyltransferase	KeggGhostKoala
110C2	7.6	2,118,500	HPS44_09625	asmA; AsmA protein	KeggGhostKoala
177E7	64.3	2,126,718	HPS44_09650	fhuE, fpvA, fptA; outer-membrane receptor for ferric coprogen and ferric-rhodotorulic acid	KeggGhostKoala
8D2	44.2	2,128,870	HPS44_09660	ychF; ribosome-binding ATPase	KeggGhostKoala
111C3	11.2	2,129,309	HPS44_09660	ychF; ribosome-binding ATPase	KeggGhostKoala
158C4	36.4	2,135,718	HPS44_09695	HAMP domain-containing protein	NCBI_PGAP
82H1	6.4	2,166,340	HPS44_09825	glycosyltransferase family 4 protein	NCBI_PGAP
76H9	6.5	2,166,466	HPS44_09825	glycosyltransferase family 4 protein	NCBI_PGAP
143G6	4.3	2,166,466	HPS44_09825	glycosyltransferase family 4 protein	NCBI_PGAP
14B2	3.9	2,171,051	HPS44_09845	glycosyltransferase family 4 protein	NCBI_PGAP
110C4	3.6	2,173,666	HPS44_09855	wbpP; UDP-N-acetylglucosamine/UDP-N-acetylgalactosamine 4-epimerase	KeggGhostKoala
47B12	20.9	2,173,750	HPS44_09855	wbpP; UDP-N-acetylglucosamine/UDP-N-acetylgalactosamine 4-epimerase	KeggGhostKoala
9E10	8.0	2,174,159	HPS44_09855	wbpP; UDP-N-acetylglucosamine/UDP-N-acetylgalactosamine 4-epimerase	KeggGhostKoala
17C7	10.2	2,174,525	HPS44_09855	wbpP; UDP-N-acetylglucosamine/UDP-N-acetylgalactosamine 4-epimerase	KeggGhostKoala
82G8	12.4	2,174,764	HPS44_09860	wbpO; UDP-N-acetyl-D-glucosamine/UDP-N-acetyl-D-galactosamine dehydrogenase	KeggGhostKoala
15F4	16.9	2,175,262	HPS44_09860	wbpO; UDP-N-acetyl-D-glucosamine/UDP-N-acetyl-D-galactosamine dehydrogenase	KeggGhostKoala
36A3	39.1	2,175,262	HPS44_09860	wbpO; UDP-N-acetyl-D-glucosamine/UDP-N-acetyl-D-galactosamine dehydrogenase	KeggGhostKoala
70E8	33.9	2,175,998	HPS44_09865	glycosyltransferase family 4 protein	NCBI_PGAP
3H9	22.0	2,176,097	HPS44_09865	glycosyltransferase family 4 protein	NCBI_PGAP
161B6	3.5	2,176,097	HPS44_09865	glycosyltransferase family 4 protein	NCBI_PGAP
85E8	7.4	2,176,277	HPS44_09865	glycosyltransferase family 4 protein	NCBI_PGAP
137H4	8.3	2,176,277	HPS44_09865	glycosyltransferase family 4 protein	NCBI_PGAP
70D2	65.4	2,201,817	HPS44_09965	glycosyltransferase family 4 protein	NCBI_PGAP
160D8	20.8	2,216,823	HPS44_10030	dcd; dCTP deaminase	KeggGhostKoala
158C9	39.8	2,328,780	HPS44_10515	heme acquisition protein HasAp	NCBI_PGAP
60F2	26.7	2,392,034	HPS44_10815	helix-turn-helix transcriptional regulator	NCBI_PGAP
159E5	4.6	2,509,162	HPS44_11345	hemK, prmC, HEMK; release factor glutamine methyltransferase	KeggGhostKoala

163A10	2.2	2,546,686	HPS44_11545	Preprotein translocase subunit SecD (SecD) (PDB:3AQP)	COG20_FUNCTION
165G7	3.2	2,546,686	HPS44_11545	Preprotein translocase subunit SecD (SecD) (PDB:3AQP)	COG20_FUNCTION
198D10	4.6	2,546,901	HPS44_11545	Preprotein translocase subunit SecD (SecD) (PDB:3AQP)	COG20_FUNCTION
28H4*	62.7	2,599,180	HPS44_11780 / HPS44_11785	Intergenic	
28H9*	44.9	2,599,180	HPS44_11780 / HPS44_11785	Intergenic	
81B9	54.5	2,680,581	HPS44_12205	hypothetical protein	NCBI_PGAP
5H7	6.2	2,700,177	HPS44_12250	circularly permuted type 2 ATP-grasp protein	NCBI_PGAP
163B6	16.7	2,700,443	HPS44_12250	circularly permuted type 2 ATP-grasp protein	NCBI_PGAP
27E11	8.2	2,706,718	HPS44_12285	ahcY; adenosylhomocysteinase	KeggGhostKoala
103E10	64.6	2,729,433	HPS44_12390	dapF; diaminopimelate epimerase	KeggGhostKoala
46E6	62.1	2,737,467	HPS44_12430 / HPS44_12435	Intergenic	
141B2*	2.9	2,745,704	HPS44_12480	lipB; lipoyl(octanoyl) transferase	KeggGhostKoala
141H3*	3.3	2,745,704	HPS44_12480	lipB; lipoyl(octanoyl) transferase	KeggGhostKoala
9A2	6.8	2,745,794	HPS44_12480	lipB; lipoyl(octanoyl) transferase	KeggGhostKoala
6A2	5.1	2,758,668	HPS44_12555	wbpD, wlbB; UDP-2-acetamido-3-amino-2,3-dideoxy-glucuronate N-acetyltransferase	KeggGhostKoala
28E2	2.9	2,759,073	HPS44_12555	wbpD, wlbB; UDP-2-acetamido-3-amino-2,3-dideoxy-glucuronate N-acetyltransferase	KeggGhostKoala
65F2	21.0	2,759,073	HPS44_12555	wbpD, wlbB; UDP-2-acetamido-3-amino-2,3-dideoxy-glucuronate N-acetyltransferase	KeggGhostKoala
118C6	2.6	2,759,073	HPS44_12555	wbpD, wlbB; UDP-2-acetamido-3-amino-2,3-dideoxy-glucuronate N-acetyltransferase	KeggGhostKoala
8A6	14.3	2,759,250	HPS44_12560	wbpE, wlbC; UDP-2-acetamido-2-deoxy-ribo-hexuluronate aminotransferase	KeggGhostKoala
57A12	5.5	2,759,604	HPS44_12560	wbpE, wlbC; UDP-2-acetamido-2-deoxy-ribo-hexuluronate aminotransferase	KeggGhostKoala
48H12	15.6	2,760,072	HPS44_12560	wbpE, wlbC; UDP-2-acetamido-2-deoxy-ribo-hexuluronate aminotransferase	KeggGhostKoala
45D1	23.7	2,760,549	HPS44_12565	glycosyltransferase	NCBI_PGAP
144C11	3.3	2,760,549	HPS44_12565	glycosyltransferase	NCBI_PGAP
28D1	3.1	2,760,640	HPS44_12565	glycosyltransferase	NCBI_PGAP
31F2	3.0	2,760,694	HPS44_12565	glycosyltransferase	NCBI_PGAP
42F4	32.5	2,760,694	HPS44_12565	glycosyltransferase	NCBI_PGAP
190D11	5.3	2,760,790	HPS44_12565	glycosyltransferase	NCBI_PGAP
106A6	4.3	2,761,091	HPS44_12565	glycosyltransferase	NCBI_PGAP
87D12	3.7	2,761,207	HPS44_12565	glycosyltransferase	NCBI_PGAP
33F6	2.7	2,761,270	HPS44_12565	glycosyltransferase	NCBI_PGAP
101A5	3.7	2,761,270	HPS44_12565	glycosyltransferase	NCBI_PGAP
105F5	4.0	2,761,270	HPS44_12565	glycosyltransferase	NCBI_PGAP
161D7	3.9	2,761,596	HPS44_12570	DegT/DnrJ/EryC1/StrS family aminotransferase	NCBI_PGAP

162A5	1.9	2,761,847	HPS44_12570	DegT/DnrJ/EryC1/StrS family aminotransferase	NCBI_PGAP
51G4	8.4	2,762,483	HPS44_12570	DegT/DnrJ/EryC1/StrS family aminotransferase	NCBI_PGAP
187H3	4.9	2,762,483	HPS44_12570	DegT/DnrJ/EryC1/StrS family aminotransferase	NCBI_PGAP
133D7	20.9	2,762,487	HPS44_12570	DegT/DnrJ/EryC1/StrS family aminotransferase	NCBI_PGAP
32D4	9.6	2,762,597	HPS44_12570	DegT/DnrJ/EryC1/StrS family aminotransferase	NCBI_PGAP
35A7	59.7	2,764,918	HPS44_12585	DUF2837 family protein	NCBI_PGAP
28E10	35.0	2,770,051	HPS44_12605	wbpI, wlbD; UDP-GlcNAc3NAcA epimerase	KeggGhostKoala
47D8	45.6	2,770,186	HPS44_12605	wbpI, wlbD; UDP-GlcNAc3NAcA epimerase	KeggGhostKoala
7D7	14.9	2,770,337	HPS44_12605	wbpI, wlbD; UDP-GlcNAc3NAcA epimerase	KeggGhostKoala
3C6	19.1	2,770,569	HPS44_12605	wbpI, wlbD; UDP-GlcNAc3NAcA epimerase	KeggGhostKoala
36D12	38.0	2,770,655	HPS44_12605	wbpI, wlbD; UDP-GlcNAc3NAcA epimerase	KeggGhostKoala
101F4*	5.4	2,770,853	HPS44_12605	wbpI, wlbD; UDP-GlcNAc3NAcA epimerase	KeggGhostKoala
101G6*	5.2	2,770,853	HPS44_12605	wbpI, wlbD; UDP-GlcNAc3NAcA epimerase	KeggGhostKoala
30D2	12.7	2,771,317	HPS44_12610	asnB, ASNS; asparagine synthase (glutamine-hydrolysing)	KeggGhostKoala
39H11	48.0	2,771,317	HPS44_12610	asnB, ASNS; asparagine synthase (glutamine-hydrolysing)	KeggGhostKoala
117A11	4.5	2,771,317	HPS44_12610	asnB, ASNS; asparagine synthase (glutamine-hydrolysing)	KeggGhostKoala
92A4	3.1	2,771,360	HPS44_12610	asnB, ASNS; asparagine synthase (glutamine-hydrolysing)	KeggGhostKoala
160C12	3.1	2,771,360	HPS44_12610	asnB, ASNS; asparagine synthase (glutamine-hydrolysing)	KeggGhostKoala
164B6	4.6	2,771,557	HPS44_12610	asnB, ASNS; asparagine synthase (glutamine-hydrolysing)	KeggGhostKoala
142F5	3.8	2,771,784	HPS44_12610	asnB, ASNS; asparagine synthase (glutamine-hydrolysing)	KeggGhostKoala
162H9	3.3	2,772,019	HPS44_12610	asnB, ASNS; asparagine synthase (glutamine-hydrolysing)	KeggGhostKoala
122F7	3.9	2,772,075	HPS44_12610	asnB, ASNS; asparagine synthase (glutamine-hydrolysing)	KeggGhostKoala
34D3	9.0	2,772,151	HPS44_12610	asnB, ASNS; asparagine synthase (glutamine-hydrolysing)	KeggGhostKoala
44F12	47.0	2,772,194	HPS44_12610	asnB, ASNS; asparagine synthase (glutamine-hydrolysing)	KeggGhostKoala
176E6	16.3	2,772,194	HPS44_12610	asnB, ASNS; asparagine synthase (glutamine-hydrolysing)	KeggGhostKoala
137E6	5.5	2,772,273	HPS44_12610	asnB, ASNS; asparagine synthase (glutamine-hydrolysing)	KeggGhostKoala
17D10	8.1	2,772,286	HPS44_12610	asnB, ASNS; asparagine synthase (glutamine-hydrolysing)	KeggGhostKoala
50H12	13.0	2,772,286	HPS44_12610	asnB, ASNS; asparagine synthase (glutamine-hydrolysing)	KeggGhostKoala
178G7*	20.8	2,772,443	HPS44_12610	asnB, ASNS; asparagine synthase (glutamine-hydrolysing)	KeggGhostKoala
178H7*	20.4	2,772,443	HPS44_12610	asnB, ASNS; asparagine synthase (glutamine-hydrolysing)	KeggGhostKoala

136E11	2.9	2,772,522	HPS44_12610	asnB, ASNS; asparagine synthase (glutamine-hydrolysing)	KeggGhostKoala
149B12	3.6	2,772,615	HPS44_12610	asnB, ASNS; asparagine synthase (glutamine-hydrolysing)	KeggGhostKoala
69C4	11.0	2,772,784	HPS44_12610	asnB, ASNS; asparagine synthase (glutamine-hydrolysing)	KeggGhostKoala
28D7	4.3	2,772,852	HPS44_12610	asnB, ASNS; asparagine synthase (glutamine-hydrolysing)	KeggGhostKoala
85F5	7.7	2,773,083	HPS44_12615	glycosyltransferase	NCBI_PGAP
176E5	30.9	2,773,083	HPS44_12615	glycosyltransferase	NCBI_PGAP
135F6	9.1	2,773,383	HPS44_12615	glycosyltransferase	NCBI_PGAP
177B5	28.4	2,773,534	HPS44_12615	glycosyltransferase	NCBI_PGAP
52C2	6.6	2,776,773	HPS44_12630	galE, GALE; UDP-glucose 4-epimerase	KeggGhostKoala
147B5	6.6	2,776,885	HPS44_12630	galE, GALE; UDP-glucose 4-epimerase	KeggGhostKoala
23F5	16.8	2,776,986	HPS44_12630	galE, GALE; UDP-glucose 4-epimerase	KeggGhostKoala
35G12	35.4	2,777,322	HPS44_12630	galE, GALE; UDP-glucose 4-epimerase	KeggGhostKoala
29H4	40.7	2,777,322	HPS44_12630	galE, GALE; UDP-glucose 4-epimerase	KeggGhostKoala
127A6	32.3	2,777,322	HPS44_12630	galE, GALE; UDP-glucose 4-epimerase	KeggGhostKoala
12G1	17.8	2,777,548	HPS44_12630	galE, GALE; UDP-glucose 4-epimerase	KeggGhostKoala
176G7	24.2	2,778,266	HPS44_12635	galE, GALE; UDP-glucose 4-epimerase	KeggGhostKoala
177H9	28.2	2,778,266	HPS44_12635	galE, GALE; UDP-glucose 4-epimerase	KeggGhostKoala
128A5	6.0	2,779,021	HPS44_12640	galE, GALE; UDP-glucose 4-epimerase	KeggGhostKoala
172B4	8.3	2,779,189	HPS44_12640	galE, GALE; UDP-glucose 4-epimerase	KeggGhostKoala
48F2	14.4	2,779,210	HPS44_12640	galE, GALE; UDP-glucose 4-epimerase	KeggGhostKoala
100G12	7.3	2,779,734	HPS44_12645	asnB, ASNS; asparagine synthase (glutamine-hydrolysing)	KeggGhostKoala
187A2	5.3	2,779,734	HPS44_12645	asnB, ASNS; asparagine synthase (glutamine-hydrolysing)	KeggGhostKoala
117C11	4.3	2,780,168	HPS44_12645	asnB, ASNS; asparagine synthase (glutamine-hydrolysing)	KeggGhostKoala
120A3	7.9	2,780,394	HPS44_12645	asnB, ASNS; asparagine synthase (glutamine-hydrolysing)	KeggGhostKoala
48F6	38.7	2,780,753	HPS44_12645	asnB, ASNS; asparagine synthase (glutamine-hydrolysing)	KeggGhostKoala
146E7	32.1	2,781,126	HPS44_12645	asnB, ASNS; asparagine synthase (glutamine-hydrolysing)	KeggGhostKoala
15B8	30.0	2,781,128	HPS44_12645	asnB, ASNS; asparagine synthase (glutamine-hydrolysing)	KeggGhostKoala
115A12	7.3	2,782,909	HPS44_12650	hypothetical protein	NCBI_PGAP
179D6	24.7	2,783,115	HPS44_12650	hypothetical protein	NCBI_PGAP
22E4	8.4	2,783,692	HPS44_12650	hypothetical protein	NCBI_PGAP
13B3	44.0	2,784,100	HPS44_12650	hypothetical protein	NCBI_PGAP
175G2	27.6	2,784,941	HPS44_12655	wzm, rfbA; lipopolysaccharide transport system permease protein	KeggGhostKoala
145B2	16.1	2,785,158	HPS44_12660	wzt, rbfB; lipopolysaccharide transport system ATP-binding protein	KeggGhostKoala
156H10	2.8	2,785,289	HPS44_12660	wzt, rbfB; lipopolysaccharide transport system ATP-binding protein	KeggGhostKoala
113G12	6.7	2,785,613	HPS44_12660	wzt, rbfB; lipopolysaccharide transport system ATP-binding protein	KeggGhostKoala
110B3	5.3	2,785,709	HPS44_12660	wzt, rbfB; lipopolysaccharide transport system ATP-binding protein	KeggGhostKoala

112B7	3.9	2,785,709	HPS44_12660	wzt, rbfB; lipopolysaccharide transport system ATP-binding protein	KeggGhostKoala
108H4	7.3	2,786,151	HPS44_12660	wzt, rbfB; lipopolysaccharide transport system ATP-binding protein	KeggGhostKoala
80G5	44.5	2,786,529	HPS44_12665	WbqC family protein	NCBI_PGAP
70E12	12.6	2,786,640	HPS44_12665	WbqC family protein	NCBI_PGAP
35F6	6.6	2,786,744	HPS44_12665	WbqC family protein	NCBI_PGAP
11D6	31.4	2,786,884	HPS44_12665	WbqC family protein	NCBI_PGAP
91C2	29.5	2,786,884	HPS44_12665	WbqC family protein	NCBI_PGAP
36G2	14.8	2,786,911	HPS44_12665	WbqC family protein	NCBI_PGAP
8D1	14.3	2,787,105	HPS44_12665	WbqC family protein	NCBI_PGAP
176H10	30.2	2,787,124	HPS44_12665	WbqC family protein	NCBI_PGAP
91G11	13.8	2,787,327	HPS44_12665	WbqC family protein	NCBI_PGAP
185B6	10.2	2,788,422	HPS44_12670	fmt; methionyl-tRNA formyltransferase	KeggGhostKoala
151F3	4.7	2,789,706	HPS44_12680	fmt; methionyl-tRNA formyltransferase	KeggGhostKoala
102B3	11.7	2,792,949	HPS44_12700	methyltransferase domain-containing protein	NCBI_PGAP
68H5	9.9	2,794,061	HPS44_12705	carB, CPA2; carbamoyl-phosphate synthase large subunit	KeggGhostKoala
88F7	18.6	2,796,173	HPS44_12715	NAD-dependent epimerase/dehydratase family protein	NCBI_PGAP
29A5	9.7	2,798,597	HPS44_12725	aminotransferase class I/II-fold pyridoxal phosphate-dependent enzyme	NCBI_PGAP
140H5	1.7	2,798,993	HPS44_12725	aminotransferase class I/II-fold pyridoxal phosphate-dependent enzyme	NCBI_PGAP
1E10	19.4	2,799,961	HPS44_12730	galE; UDP-glucose 4-epimerase	KeggGhostKoala
191C12	11.5	2,800,024	HPS44_12730	galE; UDP-glucose 4-epimerase	KeggGhostKoala
153H6	2.8	2,800,117	HPS44_12730	galE; UDP-glucose 4-epimerase	KeggGhostKoala
160B2	2.8	2,800,147	HPS44_12730	galE; UDP-glucose 4-epimerase	KeggGhostKoala
66G3	2.4	2,800,321	HPS44_12730	galE; UDP-glucose 4-epimerase	KeggGhostKoala
48H10	55.9	2,800,683	HPS44_12730	galE; UDP-glucose 4-epimerase	KeggGhostKoala
30H5	13.8	2,800,863	HPS44_12730	galE; UDP-glucose 4-epimerase	KeggGhostKoala
14E7	36.1	2,804,097	HPS44_12750	rfbC, rmlC; dTDP-4-dehydrorhamnose 3,5-epimerase	KeggGhostKoala
32E7	15.3	2,804,100	HPS44_12750	rfbC, rmlC; dTDP-4-dehydrorhamnose 3,5-epimerase	KeggGhostKoala
3E5	56.8	2,810,883	HPS44_12780	DUF502 domain-containing protein	NCBI_PGAP
145F3	6.7	2,842,256	HPS44_12925	mrcA; penicillin-binding protein 1A	KeggGhostKoala
15E10	31.4	2,843,285	HPS44_12925	mrcA; penicillin-binding protein 1A	KeggGhostKoala
143G8	3.9	2,854,132	HPS44_12975	dsbD, dipZ; thioredoxin:protein disulfide reductase	KeggGhostKoala
35D12	18.7	2,854,216	HPS44_12975	dsbD, dipZ; thioredoxin:protein disulfide reductase	KeggGhostKoala
113E4	9.2	2,854,981	HPS44_12975	dsbD, dipZ; thioredoxin:protein disulfide reductase	KeggGhostKoala
135B2	15.1	2,854,981	HPS44_12975	dsbD, dipZ; thioredoxin:protein disulfide reductase	KeggGhostKoala
119E2	22.2	2,861,605	HPS44_13035	rpmD; large subunit ribosomal protein L30	KeggGhostKoala
119C2	10.4	2,884,109	HPS44_13195	tuf; elongation factor Tu	KeggGhostKoala
73A3	6.1	2,889,219	HPS44_13220	response regulator transcription factor	NCBI_PGAP

68E11	25.0	2,899,619	HPS44_13250	rpoB; DNA-directed RNA polymerase subunit beta	KeggGhostKoala
114D3	35.4	2,927,468	HPS44_13390	parA, soj; chromosome partitioning protein	KeggGhostKoala
29H11	31.6	2,940,893	HPS44_13440	tctD; two-component system, OmpR family, response regulator TctD	KeggGhostKoala
183H11	44.9	2,955,390	HPS44_13505	katE, CAT, catB, srpA; catalase	KeggGhostKoala
81D9	65.9	3,065,806	HPS44_13975	TIGR04141 family sporadically distributed protein	NCBI_PGAP
119F6	68.3	3,074,521	HPS44_13990	DUF3893 domain-containing protein	NCBI_PGAP
184B9	4.6	3,350,745	HPS44_15235	DNA-binding beta-propeller fold protein YncE (PDB:3VGZ)	COG20_FUNCTION
37G10	9.2	3,471,979	HPS44_15800	livM; branched-chain amino acid transport system permease protein	KeggGhostKoala
121E12	42.7	3,629,053	HPS44_16505	SDR family oxidoreductase	NCBI_PGAP
117F7	57.5	3,643,924	HPS44_16575	lytic transglycosylase domain-containing protein	NCBI_PGAP
85H7	41.9	3,647,468	HPS44_16595	rubredoxin	NCBI_PGAP
162E3	10.3	3,682,613	HPS44_16755	prmA; ribosomal protein L11 methyltransferase	KeggGhostKoala
161C10	50.8	3,731,539	HPS44_16970	YbhB/YbcL family Raf kinase inhibitor-like protein	NCBI_PGAP
71G6	5.1	3,755,310	HPS44_17070	hypothetical protein	NCBI_PGAP
187F11	11.8	3,761,053	HPS44_17100	yscD, sctD, ssaD; type III secretion protein D	KeggGhostKoala
18D4	3.2	3,761,427	HPS44_17105	yscV, sctV, hrcV, ssaV, invA; type III secretion protein V	KeggGhostKoala
33B4	3.7	3,761,427	HPS44_17105	yscV, sctV, hrcV, ssaV, invA; type III secretion protein V	KeggGhostKoala
35A12	10.9	3,761,427	HPS44_17105	yscV, sctV, hrcV, ssaV, invA; type III secretion protein V	KeggGhostKoala
79G12	5.8	3,763,262	HPS44_17110	hypothetical protein	NCBI_PGAP
31E11	2.1	3,767,991	HPS44_17145	hypothetical protein	NCBI_PGAP
21F2	3.8	3,776,875	HPS44_17210	yscQ, sctQ, hrcQ, ssaQ, spaO; type III secretion protein Q	KeggGhostKoala
123A10	3.1	3,778,441	HPS44_17215	yscR, sctR, hrcR, ssaR; type III secretion protein R	KeggGhostKoala
17D3	7.5	3,778,868	HPS44_17220	yscS, sctS, hrcS, ssaS; type III secretion protein S	KeggGhostKoala
33F2	15.5	3,780,855	HPS44_17235	type III secretion system chaperone	NCBI_PGAP
163G4	2.0	3,780,855	HPS44_17235	type III secretion system chaperone	NCBI_PGAP
59D8	5.5	3,783,120	HPS44_17240 / HPS44_17245	Intergenic	
60A9	3.6	3,783,120	HPS44_17240 / HPS44_17245	Intergenic	
47F6	8.2	3,784,553	HPS44_17250	hypothetical protein	NCBI_PGAP
9F1*	5.3	3,784,871	HPS44_17255	hypothetical protein	NCBI_PGAP
9F4*	6.5	3,784,871	HPS44_17255	hypothetical protein	NCBI_PGAP
22G5	14.7	3,784,886	HPS44_17255	hypothetical protein	NCBI_PGAP
55E7	4.1	3,784,892	HPS44_17255	hypothetical protein	NCBI_PGAP
32D9	2.7	3,785,069	HPS44_17255	hypothetical protein	NCBI_PGAP
98A8	2.3	3,785,069	HPS44_17255	hypothetical protein	NCBI_PGAP
122E8	3.0	3,785,189	HPS44_17255	hypothetical protein	NCBI_PGAP
194E8	5.5	3,785,582	HPS44_17255	hypothetical protein	NCBI_PGAP

55D7	3.8	3,786,085	HPS44_17260	rsbU_P; phosphoserine phosphatase RsbU/P	KeggGhostKoala
7D11	6.5	3,786,133	HPS44_17260	rsbU_P; phosphoserine phosphatase RsbU/P	KeggGhostKoala
90B9	3.2	3,786,748	HPS44_17260	rsbU_P; phosphoserine phosphatase RsbU/P	KeggGhostKoala
97D8	2.7	3,787,979	HPS44_17260 / HPS44_17265	Intergenic	
1F11	5.8	3,787,983	HPS44_17260 / HPS44_17265	Intergenic	
113F3	4.6	3,791,898	HPS44_17275	rsbW; serine/threonine-protein kinase RsbW	KeggGhostKoala
102D2	57.4	3,791,988	HPS44_17275	rsbW; serine/threonine-protein kinase RsbW	KeggGhostKoala
45H7	4.7	3,792,298	HPS44_17275 / HPS44_17280	Intergenic	
187B10	13.7	3,792,455	HPS44_17280	STAS domain-containing protein	NCBI_PGAP
140H4	1.7	3,792,609	HPS44_17280	STAS domain-containing protein	NCBI_PGAP
178F7	25.6	3,792,834	HPS44_17285	hypothetical protein	NCBI_PGAP
39G5	6.1	3,793,106	HPS44_17285 / HPS44_17290	Intergenic	
15E9	9.9	3,806,668	HPS44_17350	dnaQ; DNA polymerase III subunit epsilon	KeggGhostKoala
113C8	17.6	3,806,668	HPS44_17350	dnaQ; DNA polymerase III subunit epsilon	KeggGhostKoala
174H6	60.0	3,812,799	HPS44_17390	gtrB, csbB; polyisoprenyl-phosphate glycosyltransferase	KeggGhostKoala
33E6	50.0	3,818,962	HPS44_17425	livM; branched-chain amino acid transport system permease protein	KeggGhostKoala
20H8	14.2	3,843,779	HPS44_17560	tolQ; biopolymer transport protein TolQ	KeggGhostKoala
177C11	58.2	3,843,892	HPS44_17565	ybgC; acyl-CoA thioester hydrolase	KeggGhostKoala
187A10	34.2	3,844,032	HPS44_17565	ybgC; acyl-CoA thioester hydrolase	KeggGhostKoala
155H9	6.2	3,856,544	HPS44_17620 / HPS44_17625	Intergenic	
64B5	40.9	3,861,199	HPS44_17655	lgt, umpA; phosphatidylglycerol---prolipoprotein diacylglyceryl transferase [EC:2.5.1.145]	KeggGhostKoala
150D4	28.5	3,873,364	HPS44_17710	hypothetical protein	NCBI_PGAP
24E5	4.9	3,951,422	HPS44_18000	LysR family transcriptional regulator	NCBI_PGAP
151D3	3.5	4,054,860	HPS44_18450	maleylacetate reductase	KeggGhostKoala
81A6	67.2	4,068,280	HPS44_18510	hypothetical protein	NCBI_PGAP
27H6	43.4	4,122,320	HPS44_18745	helix-turn-helix domain-containing protein	NCBI_PGAP
58B4	14.9	4,125,992	HPS44_18760	ettA; energy-dependent translational throttle protein EttA	KeggGhostKoala
73E11	68.8	4,130,387	HPS44_18795	minC; septum site-determining protein MinC	KeggGhostKoala
123F4	5.1	4,190,681	HPS44_19105	dapB; 4-hydroxy-tetrahydrodipicolinate reductase	KeggGhostKoala
191C6	5.5	4,190,681	HPS44_19105	dapB; 4-hydroxy-tetrahydrodipicolinate reductase	KeggGhostKoala
54D11	17.7	4,191,375	HPS44_19105	dapB; 4-hydroxy-tetrahydrodipicolinate reductase	KeggGhostKoala
141B7*	11.5	4,191,381	HPS44_19105	dapB; 4-hydroxy-tetrahydrodipicolinate reductase	KeggGhostKoala
141G7*	27.2	4,191,381	HPS44_19105	dapB; 4-hydroxy-tetrahydrodipicolinate reductase	KeggGhostKoala
161H4	3.1	4,194,332	HPS44_19125	ppnK, NADK; NAD+ kinase	KeggGhostKoala

81D2	59.2	4,279,105	HPS44_19510	hypothetical protein	NCBI_PGAP
181G3	9.8	4,282,155	HPS44_19520	pnp, PNPT1; polyribonucleotide nucleotidyltransferase	KeggGhostKoala
112F4	7.0	4,293,653	HPS44_19580	secG; preprotein translocase subunit SecG	KeggGhostKoala
83G12	12.1	4,293,932	HPS44_19580	secG; preprotein translocase subunit SecG	KeggGhostKoala
13B11	13.9	4,466,735	HPS44_20275 / HPS44_20280	Intergenic	
110H6	50.0	4,470,327	HPS44_20295	msrQ; methionine sulfoxide reductase heme-binding subunit	KeggGhostKoala
88D3	57.7	4,472,805	HPS44_20315	uncharacterized protein	KeggGhostKoala
131D7	60.8	4,477,940	HPS44_20345 / HPS44_20350	Intergenic	
83E4	5.1	4,479,708	HPS44_20355	hypothetical protein	NCBI_PGAP
90A7	3.8	4,479,708	HPS44_20355	hypothetical protein	NCBI_PGAP
103C8	7.1	4,479,738	HPS44_20355	hypothetical protein	NCBI_PGAP
105C12	3.7	4,480,596	HPS44_20365	rseA; sigma-E factor negative regulatory protein RseA	KeggGhostKoala
162H3	3.3	4,480,596	HPS44_20365	rseA; sigma-E factor negative regulatory protein RseA	KeggGhostKoala
2D8	4.9	4,480,887	HPS44_20365	rseA; sigma-E factor negative regulatory protein RseA	KeggGhostKoala
95A3	3.4	4,481,174	HPS44_20370	rseB; sigma-E factor negative regulatory protein RseB	KeggGhostKoala
106C8	3.6	4,481,174	HPS44_20370	rseB; sigma-E factor negative regulatory protein RseB	KeggGhostKoala
109B3	4.2	4,481,174	HPS44_20370	rseB; sigma-E factor negative regulatory protein RseB	KeggGhostKoala
139H3	2.6	4,481,174	HPS44_20370	rseB; sigma-E factor negative regulatory protein RseB	KeggGhostKoala
68A6	6.6	4,481,192	HPS44_20370	rseB; sigma-E factor negative regulatory protein RseB	KeggGhostKoala
90H1	3.2	4,481,192	HPS44_20370	rseB; sigma-E factor negative regulatory protein RseB	KeggGhostKoala
94F5*	3.0	4,481,192	HPS44_20370	rseB; sigma-E factor negative regulatory protein RseB	KeggGhostKoala
94G8*	4.0	4,481,192	HPS44_20370	rseB; sigma-E factor negative regulatory protein RseB	KeggGhostKoala
122C8	2.0	4,481,420	HPS44_20370	rseB; sigma-E factor negative regulatory protein RseB	KeggGhostKoala
169G5	4.5	4,481,420	HPS44_20370	rseB; sigma-E factor negative regulatory protein RseB	KeggGhostKoala
114A7	2.9	4,481,849	HPS44_20370	rseB; sigma-E factor negative regulatory protein RseB	KeggGhostKoala
135F7	2.2	4,481,912	HPS44_20370	rseB; sigma-E factor negative regulatory protein RseB	KeggGhostKoala
107G1	8.4	4,481,999	HPS44_20370	rseB; sigma-E factor negative regulatory protein RseB	KeggGhostKoala
48C2	38.2	4,482,080	HPS44_20375	degP, htrA; serine protease Do	KeggGhostKoala
137C4	61.2	4,483,265	HPS44_20375	degP, htrA; serine protease Do	KeggGhostKoala
102F11	9.8	4,484,006	HPS44_20380	lepA; GTP-binding protein LepA	KeggGhostKoala
136B8	35.2	4,484,402	HPS44_20380	lepA; GTP-binding protein LepA	KeggGhostKoala
155H10	4.3	4,485,203	HPS44_20380	lepA; GTP-binding protein LepA	KeggGhostKoala
117G3	63.7	4,505,053	HPS44_20480	DUF2325 domain-containing protein	NCBI_PGAP
195D11	11.5	4,523,683	HPS44_20565	elaA; ElaA protein	KeggGhostKoala

46B2	61.5	4,555,988	HPS44_20710	mdh; malate dehydrogenase	KeggGhostKoala
97E12	22.0	4,577,672	HPS44_20795 / HPS44_20800	Intergenic	
48H11	35.7	4,582,176	HPS44_20810	DLD, lpd, pdhD; dihydrolipoamide dehydrogenase	KeggGhostKoala
114B7	66.0	4,604,190	HPS44_20895	phbB; acetoacetyl-CoA reductase	KeggGhostKoala
119E12	62.3	4,739,421	HPS44_21485	infB, MTIF2; translation initiation factor IF-2	KeggGhostKoala
66A10	5.0	4,740,145	HPS44_21485	infB, MTIF2; translation initiation factor IF-2	KeggGhostKoala
77E2	46.2	4,740,145	HPS44_21485	infB, MTIF2; translation initiation factor IF-2	KeggGhostKoala
193F5	5.3	4,742,940	HPS44_21490 / HPS44_21495	Intergenic	
113G8	16.6	4,824,207	HPS44_21875	hemY; HemY protein	KeggGhostKoala
114C11	31.9	4,876,328	HPS44_22130	hypothetical protein	NCBI_PGAP
159G12	10.5	4,890,361	HPS44_22215	GGDEF domain-containing protein	NCBI_PGAP
110F2	6.0	4,908,301	HPS43_22860 / HPS43_22865	Intergenic	
66F8	28.6	4,915,830	HPS44_22355	exbB; biopolymer transport protein ExbB	KeggGhostKoala
24A2	9.0	4,950,407	HPS44_22520	phoU; phosphate transport system protein	KeggGhostKoala
26G2	6.5	4,950,407	HPS44_22520	phoU; phosphate transport system protein	KeggGhostKoala
83F2	15.0	4,951,026	HPS44_22520	phoU; phosphate transport system protein	KeggGhostKoala
10B11	11.5	4,969,335	HPS44_22575	glycosyltransferase family 2 protein	NCBI_PGAP
183B9	22.7	4,969,787	HPS44_22580	glycosyltransferase family 4 protein	NCBI_PGAP
169D8	35.3	5,028,516	HPS44_22825	trxA; thioredoxin 1	KeggGhostKoala
186E12	68.6	5,029,107	HPS44_22830	parE; topoisomerase IV subunit B	KeggGhostKoala
111D11	15.6	5,074,021	HPS44_23040	hflK; modulator of FtsH protease HflK	KeggGhostKoala
35H3	7.0	5,082,422	HPS44_23070 / HPS44_23075	Intergenic	
110G9	22.7	5,097,897	HPS44_23140	tadB; tight adherence protein B	KeggGhostKoala
136F2	53.2	5,109,233	HPS44_23190	EAL domain-containing protein	NCBI_PGAP
40H11	45.5	5,109,605	HPS44_23190	EAL domain-containing protein	NCBI_PGAP
134E1	42.5	5,110,255	HPS44_23190 / HPS44_23195	Intergenic	
73F9	54.8	5,110,278	HPS44_23190 / HPS44_23195	Intergenic	
18E8	32.4	5,122,901	HPS44_23230	Ca2+-binding protein, RTX toxin-related (PDB:1AF0)	COG20_FUNCTION
26F2	14.0	5,132,467	HPS44_23305	lapE; outer membrane protein, adhesin transport system	KeggGhostKoala
185E1	59.9	5,276,139	HPS44_24040	K00666; fatty-acyl-CoA synthase	KeggGhostKoala
69F4	33.8	5,514,006	HPS44_25140	K00666; fatty-acyl-CoA synthase	KeggGhostKoala
50B2	29.1	5,526,029	HPS44_25185	PAS domain S-box protein	NCBI_PGAP
90E7	46.9	5,526,483	HPS44_25185	PAS domain S-box protein	NCBI_PGAP
46D1	28.5	5,526,687	HPS44_25185	PAS domain S-box protein	NCBI_PGAP
141G11	2.5	5,527,009	HPS44_25185	PAS domain S-box protein	NCBI_PGAP
155D4	38.4	5,607,906	HPS44_25555	fliS; flagellar secretion chaperone FliS	KeggGhostKoala
156D10	7.5	5,607,906	HPS44_25555	fliS; flagellar secretion chaperone FliS	KeggGhostKoala
112C4	55.0	5,608,083	HPS44_25555	fliS; flagellar secretion chaperone FliS	KeggGhostKoala
69B3	7.6	5,617,962	HPS44_25610	purF, PPAT; amidophosphoribosyltransferase	KeggGhostKoala

150F6	27.8	5,625,515	HPS44_25640	hypothetical protein	NCBI_PGAP
165E9	19.6	5,652,274	HPS44_25785	type II toxin-antitoxin system RatA family toxin	NCBI_PGAP
70D11	46.6	5,735,646	HPS44_26230	AAA family ATPase	NCBI_PGAP
67C2	15.9	5,750,917	HPS44_26300	ldcA; muramoyltetrapeptide carboxypeptidase	KeggGhostKoala
53E5	12.6	5,751,046	HPS44_26300	ldcA; muramoyltetrapeptide carboxypeptidase	KeggGhostKoala
14D1	11.3	5,751,241	HPS44_26300	ldcA; muramoyltetrapeptide carboxypeptidase	KeggGhostKoala
62A7	5.4	5,751,241	HPS44_26300	ldcA; muramoyltetrapeptide carboxypeptidase	KeggGhostKoala
156E9	33.3	5,971,841	HPS44_27310	parS; two-component system, OmpR family, sensor kinase ParS	KeggGhostKoala
33A11	67.1	6,211,977	HPS44_28430	hypothetical protein	NCBI_PGAP
10F2	60.7	6,390,695	HPS44_29175	transposase	NCBI_PGAP

* Indicates possible strain duplication due to cross-contamination during processing of the same plate. We considered insertions that mapped to the same nucleotide but isolated from separate transformations as independent events.

Movie S1. Live imaging of J774a.1 cells infected with GN050 pDBD2*sfGFP* at an MOI:5 for 30 min followed by treatment with antibiotics to select for intracellular bacteria. After 30 min of treatment, the infection was replaced with antibiotic-free medium containing 0.36 µl/ml PI and imaged every 15 min for 20 h. Chloramphenicol (10 µg/ml) was supplemented into the infection to promote plasmid maintenance and *sfGFP* expression. Scale = 25 µm.

Movie S2. FITC and dsRED channels extracted from supplemental movie 1 to emphasize bacterial proliferation in relation to cytotoxicity. Scale = 25 µm.

Movie S3. Live imaging of J774a.1 cells infected with GN050 pDBD2*sfGFP* at an MOI:5 for 30 min followed by treatment with antibiotics to select for intracellular bacteria. After 30 min of treatment, the infection medium was replaced with medium containing 50 µg/ml gentamicin, 10 µg/ml polymyxin B, 0.36 µl/ml PI and imaged every 15 m for 20 h. Chloramphenicol (10 µg/ml) was supplemented into the infection to promote plasmid maintenance and *sfGFP* expression. Scale = 25 µm.

Movie S4. FITC and dsRED channels extracted from supplemental movie 2 to emphasize bacterial proliferation in relation to cytotoxicity. Scale = 25 µm.

SUPPLEMENTAL METHODS

Construction of pDBD2*sfGFP* and Modification of *himar1*

Antibiotic resistance is problematic in *Achromobacter* species. Initial characterization of antibiograms suggested that chloramphenicol could be useful at high concentrations (100-200 µg/ml) for selection in several clinical isolates from the Medical College campus, including GN050. As IncP plasmids appear to replicate in *Achromobacter* (4,5), we constructed pDBD2 utilizing the broad host range vector pCM62 (2). The *CmΩ* (6) interposon was cloned into the EcoRI site of pCM62 and chloramphenicol resistant clones were selected. To reduce the size of the plasmid the genes encoding tetracycline resistance (*tetA* and *tetR*) and terminator sequences were removed using the Q5 mutagenesis kit (New England Biolabs). This plasmid is called pDBD1. Plasmid DBD2 was constructed from pDBD1 by cloning the 492 bp 30S ribosomal protein S21 promoter region into the KpnI/SacI sites of pDBD1. *sfGFP* was amplified from pBAD*sfGFP* (gift from the Mekalanos laboratory) and cloned into the EcoRI site. All cloning steps utilized the Gibson assembly Master Mix (New England Biolabs) and primers purchased from Integrated DNA Technologies (IDT). *himar1* was modified to express chloramphenicol resistance as a single copy gene after transposition to the genome of GN050. Briefly, the 30S ribosomal protein S21 promoter region and *CmΩ* open reading frame were constructed as a cassette in a potential allelic replacement vector, pDBD4. The cassette was amplified from pDBD4 and inserted into the NheI site of *himar1*. All clones and plasmids were verified by double strand DNA sequence analysis.

Transposon mutagenesis screen

To identify GN050 *himar1* insertion mutants that were unable to induce cytotoxicity in J774a.1 macrophages, a crystal violet-based screen was optimized using a 96-well plate format. J774a.1 macrophages were seeded at a density of 2.9×10^4 cells per well. GN050 *himar1* insertion mutants that were recovered from LB plates supplemented with 200 µg/ml chloramphenicol were picked into

individual wells of a 96-well plate containing 200 µl of DMEM. After suspension of the colony, 5 µl of bacterial culture was added to an individual well of seeded J774a.1 macrophages (MOI of 50-200) and subject to a light centrifugation of 600 x *g* for 5 min to synchronize the infection. Each well of the bacterial suspensions were replica plated using a 96-prong tamper onto LB agar containing 100 µg/ml chloramphenicol. These would be used for isolating downstream mutants of interest. After 4 h, wells were washed with HBSS (Gibco) and stained with 3% crystal violet in 5% methanol 5% ethanol 5% isopropanol for 5 min at room temperature (RT). Each well was then washed with deionized water to remove excess crystal violet followed by an image capture to document the staining pattern of each well.

Wells that retained a majority of crystal violet staining were thought to be infected with a GN050 mutant that was non-toxic to J774a.1 cells. These selected wells were paired with mirrored replica plate mutants that grew on medium containing 100 µg/ml chloramphenicol, confirming the presence of the selectable marker. Each selected GN050 mutant was struck on LB for further analysis. Mutants that grew in a comparable manner to WT GN050 on LB were processed in a secondary screen with a standardized MOI. Here, J774a.1 cells seeded at 1.75 x 10^5 cells per well in 24-well plates were infected with WT GN050 and mutant derivatives at an MOI of 10:1 for 6-8 h after a centrifugation of 600 x *g* for 5 min. To quantify cytotoxicity, adenylate kinase (AK) release assays were carried out as previously described (1). Genome location of *himar1* insertions were determined for GN050 mutants that were at 70% or below of WT toxicity in the secondary screen.

Sequencing and functional annotation of *himar1* insertion mutants

Clones, possessing similar growth properties to GN050 but defective for cytotoxicity in primary and secondary screens, were processed for genomic DNA isolation using the DNeasy ultraclean microbial kit according to manufacturers' instructions (Qiagen). One microgram of genomic DNA was digested with NotI in a 20 µl reaction. Restriction endonuclease reactions were heated to 65˚C for 20 min to inactivate NotI and 50 ng of DNA was ligated to rescue plasmid containing the R6Kori from

himar1. Ligations were transformed into DH5α λpir and selected on medium with 30 µg/ml chloramphenicol. The resulting colonies were screened for kanamycin resistance. Plasmid DNA was isolated from colonies expressing both chloramphenicol and kanamycin resistance and subjected to DNA sequence analysis utilizing primers that bind to each end of *himar1*.

Quantitative reverse transcription-PCR (RT-qPCR)

GN050 and derived strains were grown in LB to an OD_{600} of 0.8-1.0 and 3 mls of culture were harvested by centrifugation at 16,100 x g for 5 min at RT. The supernatant was removed and the resulting pellet was suspended in 500 µl of TRIzol reagent (Ambion). For every 500 µl of TRIzol sample, 100 µl was mixed in a phasemaker tube (Invitrogen) and subject to centrifugation at 16,100 x g for 30 min at 4ºC. Total RNA was extracted using the RNeasy plus universal minikit (Qiagen) and quantified using a Qubit fluorometer. Using 1 µg of total RNA, DNase and reverse transcriptase reactions were performed using SuperScript IV VILO (Thermo Scientific) per the manufacturers' instructions. To detect any contaminating DNA, reactions excluding reverse transcriptase were performed for each group. Approximately 75 ng of cDNA was used in each qPCR experiment performed in technical triplicates using primers (0.5 µM) generated by the IDT PrimerQuest tool. Primers and template were mixed with nuclease-free water and 10 µl of 2X SsoAdvanced Universal SYBR green Supermix (Bio-Rad). qPCR was performed on a CFX Connect real-time system (Bio-Rad) and analyzed using CFX Maestro software (v4.1).

Functional annotation of insertion mutants

Transposon insertions that mapped to an ORF were assigned functional annotation identifiers. *Ax* GN050 genome containing ORF identifiers (FASTAS) were exported from the NCBI genome database (CP053617) and converted into an anvi'o (v7, "Hope")-useable contig database file (.db). Functional annotations were identified against COG and PFAM databases. Protein sequences

(.fa) were exported from anvi'o and imported into GhostKOALA (Kanehisa Laboratories) for KEGG annotation.

Pangenomic and phylogenomic analyses

Pangenomic analysis of *Ax* strains were performed using the anvi'o suite (v7, "Hope"). *Ax* genomes for 88 isolates were obtained from NCBI RefSeq databases (FASTAS) and were used to generate the anvi'o-readable contigs database file (.db; 'anvi-generate-contigs-database'). A single storage database was generated for the 88 genomes by using 'anvi-gen-genomes-storage' with the command '—gene-caller NCBI_PGAP'. The pangenome analysis was performed using 'anvi-pan-genome' under default settings (Use DIAMOND [7], '—minbit = 0.5' and '—mcl-inflation = 2') and resulting protein clusters were visualized using 'anvi-display-pan'. The figure was exported (.svg) and manipulated for aesthetics using inkscape (v1.0).

To determine the phylogenetic relationship of the 88 *Ax* isolates, an anvi'o (v7, "Hope") workflow for phylogenomics was used. A new bin collection in 'anvi-display-pan' was generated to identify core single-copy genes (SCG's) across *Ax* genomes. Because the strains were highly similar, custom homogeneity indices (Min number of genomes gene cluster occurs = 88, Max number of genes from each genome = 1, Max functional homogeneity index = 0.95, Min geometric homogeneity index = 0.9) were applied to identify 77 SCG's Alignments from the pangenomic analysis described above and using the bin collection with 77 SCG's were exported (.fa) using 'anvi-get-sequences-for-gene-clusters' with the command '—concatenate-gene-clusters' and '—max-num-genes-from-each-genome = 1'. A phylogenetic tree (maximum-likelihood method) was generated using 'anvi-gen-phylogenomic-tree' and visualized in 'anvi-interactive'. The tree was exported into a Newick file (.nwk) then rooted by midpoint and visualized in FigTree (v1.4.4). The figure was exported (.svg) and manipulated for aesthetics using inkscape (v1.0).

Microscopy and image processing

Microscopy images were generated using a Nikon Eclipse Ti inverted microscope system equipped with a CoolSNAP ES2 charge-coupled-device (CCD) camera (Photometrics) with NIS-Elements AR imaging software (v5.2). For binding assays, images were captured using a Nikon 10X (Plan Apo 0.3 NA) or 100X (Plan Apo 1.4 NA oil) lens. For live imaging, images were captured in intervals using a Nikon 10X (Plan Fluor 0.3 NA) or 60X (Plan Apo 1.4 NA oil) lens. Infections were carried out on a motorized stage inside a Tokei Hit environmental chamber temperature-controlled at 37ºC and 5% Clinical Blood Gas (Airgas). In both cases image intensity of propidium iodide (cytotoxicity assays), GFP signal (bacterial proliferation assays), or Alexa Fluor-488 signal (binding assays) was quantified using CellProfiler (v3.1.5) using the 'Measure-Image-Intensity' module.

Transmission election microscopy (TEM)

Cells in culture dishes were fixed in mixture of 2.5% glutaraldehyde + 2% paraformaldehyde in 100mM sodium cacodylate buffer pH 7.4 (8) for 1 h at RT. Following fixation the cells were washed 3x5 min in cacodylate buffer then post fixed in reduced 1% osmium tetroxide (9) for 2 h on ice. Cells were scraped and the suspension was centrifuged at 8,000 x g for 10 min. The cell pellet was kept intact during washing (3 x 5 min) with distilled water then dehydrated through a graded methanol series followed by 2 x 10 min washes in acetonitrile before infiltrating with EMBed 812 epoxy resin and polymerization overnight at 70ºC. Ultrathin sections (70 nm) were cut, stained with uranyl acetate and lead citrate, and examined in a JEOL 1400 transmission electron microscope. Images were recorded using an AMT "nanosprint 12" digital camera.

Western blotting analyses

To prepare bacterial supernatants, GN050 and derived strains were grown in Tryptic Soy Broth (TSB) dialysate medium overnight at 37ºC shaking at 250 rotations per minute (rpm). Cultures were subject to centrifugation at 3,823 x g for 10 min at RT. Supernatants were harvested and filter

sterilized through a 0.2 µm filter. Approximately 9.6 ml of supernatant was mixed with 12 ml of saturated ammonium sulfate solution (Thermo Scientific) and placed on ice for 3 h. Precipitated material was collected by centrifugation at 31,209 x g for 30 min at 4ºC followed by another brief centrifugation (31,209 x g for 5 min at 4ºC) to remove as much supernatant as possible. Pellets were suspended in 240 µl 1x SDS loading buffer (0.1 M dithiothreitol) and boiled for 5 min before loading on 8% acrylamide, SDS gels.

For *artA* expression analyses, *E. coli* BL21 (DE3) pLysS pET15b*artA* was grown in 25 ml Terrific Broth (TB) shaking at 250 rpm to an OD_{600} of 0.5-0.7. Induction of gene expression was initiated by addition of isopropyl β - d-1 thiogalactopyranoside (IPTG; 0.5 mM final concentration) or mock buffer (uninduced) and incubated for an additional 3 h. Aliquots of 40 µl from growing cultures were mixed with 10 µl of 5x SDS loading buffer (0.1 M dithiothreitol).

Antigen preparations described above were analyzed by SDS-PAGE (8% acrylamide gel) and probed with CF patient serum (1:500, overnight, 4ºC [Cystic Fibrosis Foundation]), anti-His antibody (1:10,000, overnight, 4ºC [GenScript]), and either horse radish peroxidase (HRP)-conjugated anti-human IgG (1:20,000, 2 h, RT [Thermo Scientific]) or HRP-conjugated anti-mouse IgG (1:10,000, 2 h, RT [Invitrogen]). Coomassie stained gels and western blots were imaged using a ChemiDoc Touch Gel imaging System (Bio-Rad).

Figures and statistical analyses

Figures were generated and statistical analyses were performed using python (v3.8.5) in a JupyterLab notebook environment and figures were modified using Inkscape software (v1.1). When applicable, one-way or two-way ANOVA statistical tests were performed in combination with Tukey's *post hoc* analysis. Generally, cutoff for significance was at least $p<.001$ unless otherwise specified. For each method, the statistical analysis used is indicated in the figure legend.

SUPPLEMENTAL REFERENCES

1. Pickrum AM, DeLeon O, Dirck A, Tessmer MH, Riegert MO, Biller JA, Ledeboer NA, Kirby JR, Frank DW. 2020. *Achromobacter xylosoxidans* cellular pathology is correlated with activation of a type III secretion system. Infect Immun 88:e00136-20.
2. Marx CJ, Lidstrom ME. 2001. Development of improved versatile broad-host-range vectors for use in methylotrophs and other Gram-negative bacteria. Microbiol 147:2065-2075.
3. Rubin EJ, Akerley BJ, Novik VN, Lampe DJ, Husson RN, Mekalanos JJ. 1999. *In vivo* transposition of mariner-based elements in enteric bacteria and mycobacteria. Proc Natl Acad Sci USA 96:645-50.
4. Jencova V, Strnad H, Chodora Z, Ulbrich P, Vlcek C, Hickey WJ, Paces V. 2008. Nucleotide sequence, organization and characterization of the (halo)aromatic acid catabolic plasmid pA81 from *Achromobacter xylosoxidans* A8. Res Microbiol 159:118-127.
5. Ciok A, Dziewit L, Grzesiak, Budzik JK, Gorniak D, Zdanowski MK, Bartosik D. 2016. Identification of miniature plasmids in psychrophilic Artic bacteria of the genus *Variovorax*. FEMS Microbiol Ecol 92:1-9.
6. Fellay R, Frey J, Krisch H. 1987. Interposon mutagenesis of soil and water bacteria: a family of DNA fragments designed for in vitro insertional mutagenesis of Gram-negative bacteria. Gene 52:147-154.
7. Buchfink B, Zie C, Huson DH. 2015. Fast and sensitive protein alignment using DIAMOND. Nature Methods 12:59-60.
8. Karnovsky MJ. 1965. A formaldehyde-glutaraldehyde fixative of high osmolality for use in electron-microscopy. J Cell Biol 27:137-138A.
9. McDonald K. 1984. Osmium ferricyanide fixation improves microfilament preservation and membrane visualization in a variety of animal cell types. J Ultrastruct Res 86:107-118.
